# Anti-Leukaemic Activity of Rilpivirine Is Mediated by Aurora A Kinase Inhibition

**DOI:** 10.3390/cancers15041044

**Published:** 2023-02-07

**Authors:** Saiful Islam, Muhammed H. Rahaman, Mingfeng Yu, Benjamin Noll, Jennifer H. Martin, Shudong Wang, Richard Head

**Affiliations:** 1 Drug Discovery and Development, Clinical and Health Sciences, University of South Australia, Adelaide, SA 5000, Australia; 2 Centre for Human Drug Repurposing and Medicines Research, University of Newcastle, Newcastle, NSW 2305, Australia

**Keywords:** repurposing, rilpivirine, targeted therapy, Aurora A kinase, cancer, leukaemia

## Abstract

**Simple Summary:**

Rilpivirine is an anti-viral drug used for treating human immunodeficiency virus (HIV) patients. In a recent study, we demonstrated that rilpivirine inhibited Aurora A, a protein kinase that mainly regulates the mitotic events of the cell-division cycle. Importantly, Aurora A kinase is aberrantly expressed in multiple cancer types, including acute myeloid leukaemia (AML). In the current report, we have shown that rilpivirine suppressed the growth of all AML cells investigated, consistent with its Aurora A kinase inhibition. These findings open the way for exploring this anti-viral drug as a candidate for an anti-cancer drug against AML in further pre-clinical studies.

**Abstract:**

Acute myeloid leukaemia (AML) affects predominantly elderly people and has an incidence of 1% of all cancers and 2% of all cancer deaths. Despite using intensive chemotherapy and allogeneic stem cell transplantation, the treatment options for AML remain open for innovation. Thus, there is a need to explore alternative therapies such as less toxic targeted therapies in AML. Aurora A kinase is a well-established target for the treatment of various cancers, including AML. This kinase plays a pivotal role in the cell-division cycle, particularly in different stages of mitosis, and is also involved in many other cellular regulatory processes. In a previous study, we demonstrated that the anti-viral drug rilpivirine is an Aurora A kinase inhibitor. In the current study, we have further explored the selectivity of rilpivirine for Aurora A kinase inhibition by testing this drug against a panel of 429 kinases. Concurrently, we demonstrated that rilpivirine significantly inhibited the proliferation of AML cells in a time- and concentration-dependent manner that was preceded by G_2_/M cell-cycle arrest leading to the induction of apoptosis. Consistent with its kinase inhibitory role, rilpivirine modulated the expression of critical proteins in the Aurora A kinase-signalling pathway. Importantly, orally administered rilpivirine significantly inhibited tumour growth in an HL-60 xenograft model without showing body weight changes or other clinical signs of toxicity. Furthermore, rilpivirine enhanced the anti-proliferative efficacy of the conventional anti-leukaemic chemotherapeutic agent cytarabine. Collectively, these findings provide the stimulus to explore further the anti-leukaemic activity of the anti-viral drug rilpivirine.

## 1. Introduction

Acute myeloid leukaemia (AML) is a haematological malignancy characterised by the uncontrolled clonal expansion of immature blasts in the myeloid lineage that interferes with the normal development of red blood cells, platelets, and white blood cells [1]. It is a disease principally of older adults, with a five-year survival rate of approximately 30% [2]. The current curative treatments for AML include intensive induction chemotherapy, consolidation chemotherapy, and allogeneic stem cell transplantation, but these treatment modalities may offer limited survival benefits [3,4]. In general, chemotherapeutics are effective in inhibiting the proliferation of unwanted neoplastic cells in most subtypes of AML. However, due to a commonality in cell-cycle regulatory pathways in all proliferating cells, chemotherapeutic agents may carry the risk of toxicity due to damage to normal cells. Moreover, those who can tolerate AML treatments may experience a high burden of side effects, bringing into focus again the need for less toxic entities [5]. Thus, a key challenge is the need to develop less toxic therapies using the understanding of molecular aberrations in AML [6]. Additionally, it is hoped that combinations of novel targeted agents will decrease the reliance on traditional cytotoxic agents with a reduction in side effects while maintaining efficacy [7]. All these considerations reflect the need to explore targeted therapies in AML.

The fundamental purpose of targeted therapies is to inhibit the growth of tumour cells with a minimal cytotoxic penalty for non-tumour cells, particularly those healthy cells that undergo significant replication as part of cellular haemostasis. What has made this concept suitable for empirical testing has been the recognition that a malignant phenotype can be dependent on only one or a few genes [8]. It is noteworthy that haematopoietic cells featured strongly in the development of this concept. It was shown that only one genetic aberration, the *BCR-ABL* translocation, is central to the pathogenesis of chronic myeloid leukaemia, which led to the development and subsequent approval of imatinib, a BCR-ABL kinase inhibitor for treating this cancer type [8]. Subsequent experimentation highlighted the crucial role of protein kinases in regulating cell-cycle events in normal cell division as well as in abnormal hyperplastic states. In this regard, the role of oncogenic kinases has been described with specific kinases reflecting a druggable target class [8]. Moreover, the promiscuous nature of kinases renders these specific enzymes attractive targets in the drug repurposing approach in oncology [9,10].

In an earlier study, we conducted an in silico and linked in vitro series of repurposing experiments to explore if existing approved therapeutic agents, not known to be kinase inhibitors, could modify the activities of key oncogenic kinases [11]. In doing so, we identified that the non-nucleoside reverse transcriptase inhibitor rilpivirine is an Aurora A kinase inhibitor with low sub-micromolar potency [11]. Aurora A is an oncogenic kinase that regulates mitotic entry, centrosome maturation, centrosome separation, and spindle formation and is critical for DNA-damaged induced checkpoint recovery [12]. The biology of Aurora A, its association with cancer, as well as the status of the specifically synthesised Aurora A kinase inhibitors have been reviewed in detail elsewhere [12,13,14]. To date, at least eleven Aurora A-specific or pan-aurora kinase inhibitors have been evaluated in clinical trials [13]. Notably, an aberrant expression or amplification of the *AURKA* gene (that codes Aurora A kinase) is common in various cancers, including AML [15,16,17]. Aurora A expression was found to be markedly increased in 65 of 98 (66%) AML cells from de novo AML patients, but its expression was negligible in normal bone marrow specimens [4]. Previous studies also reported promising anti-leukaemic activity of the established Aurora A kinase inhibitors, including alisertib and ENMD-2076 in AML [18,19,20]. These settings prompted us to evaluate the pre-clinical efficacy of rilpivirine in AML cells.

In this study, we explored the pre-clinical potential of rilpivirine as a possible therapeutic option for the treatment of AML. We evaluated the impact of rilpivirine on the proliferation of AML cells and studied the cellular mechanisms of its anti-leukaemic action. In addition, we assessed the in vivo safety and efficacy of rilpivirine in a xenograft mouse model. Finally, we evaluated the combinatory effects of rilpivirine and the standard anti-leukaemic chemotherapy, cytarabine. To the best of our knowledge, this is the first illustration of the efficacy of the anti-viral drug rilpivirine in inhibiting the proliferation of AML cells in a manner consistent with Aurora A kinase inhibition.

## 2. Materials and Methods

### 2.1. Chemicals

Rilpivirine, etravirine, and cytarabine were purchased from MedChem Express (Monmouth Junction, NJ, USA) or Targetmol (Boston, MA, USA). All purchased drugs had reported purity of ≥95% from the commercial suppliers. Resazurin and dimethyl sulfoxide (DMSO) were purchased from Sigma-Aldrich (Castle Hill, NSW, Australia). All drug solutions were prepared as 10 mM stocks with 100% DMSO and stored at −20 °C.

### 2.2. Kinase Assay

Kinome-wide selectivity of rilpivirine was measured using the kinase screening and profiling service of Eurofins Scientific (Le bois l’Evêque 86600. Celle-Lévescault, France) and *K*_i_ values were determined using radioisotope-based assays by Reaction Biology Corporation (Malvern, PA, USA). The experimental details of Eurofin Scientific and Reaction Biology services for relevant assays are available at https://www.eurofinsdiscoveryservices.com/services/in-Vitro-assays/kinases/kinase-profiler (accessed on 7 July 2021) and https://www.reactionbiology.com (accessed on 7 July 2021), respectively. *K*_i_ values were estimated from the corresponding IC_50_ values using the Cheng–Prusoff equation [21] and the *K*_m_ (ATP) of individual kinase was used for IC_50_ determination.

### 2.3. Cell Culture

All leukaemia cell lines (HL60, NB4, K-562, U-937, and KG1a) were cultured in RPMI 1640 medium (Gibco, VIC, Australia) supplemented with 10% foetal bovine serum (Thermo Fisher Scientific, VIC, Australia). Cell lines were kindly provided by Prof. R. D’Andrea (Acute Leukemia Laboratory, University of South Australia, Australia), which were originally sourced from DSMZ (Braunschweig, Germany). Low-passage cells were thawed for culturing and the cell lines were regularly tested for mycoplasma with the MycoAlert™ mycoplasma detection kit (Lonza, Derrimut, VIC, Australia).

### 2.4. Cell Viability Assay

Cell viability was determined by a resazurin-based assay as described previously [22]. Briefly, 5 × 10^3^ cells/well were seeded into 96-well plates (Corning Inc., Corning, NY, USA) in 180 µL of RPMI 1640 medium and incubated overnight at 37 °C with 5% CO_2_. Next day, cells were treated with rilpivirine after serially diluted (1:2 dilution for final concentrations ranging from 0.625 µM to 40 µM) or DMSO (0.1%) for specified time periods (24, 48, or 72 h). At the end of the treatment period, 20 µL of resazurin (0.1 mg/mL in PBS) was added per well and incubated for further 4 h. Fluorescence intensities were recorded at 570 nm (excitation)/585 nm (emission) using an EnVision^®^ multilabel plate reader (PerkinElmer, MA, USA). Drug concentrations required for cell viability reduction by 50% (GI_50_) compared with untreated (DMSO vehicle-treated only) control were determined by a non-linear regression model using GraphPad Prism software version 7.02 (La Jolla, CA, USA).

### 2.5. Colony Formation Assay

For colony formation assay, 2 × 10^3^ cells/well (in 6-well plates) were treated for 10 days with rilpivirine (2.5, 5, or 10 µM) or DMSO (0.1%) and incubated with 1.5 mL of cytokine-free H4230 medium (STEMCELL Technologies, Inc., Vancouver, BC, Canada). After the incubation period, cells were stained with 300 µL/well of 3-(4,5-dimethylthiazol-2-yl)-2,5-diphenyltetrazolium bromide solution (0.5 mg/mL in PBS) (Sigma-Aldrich, St Louis, MO, USA) and incubated for 1 h. Colonies were then counted on an inverted microscope.

### 2.6. Cell Cycle Analysis

To determine the effect of rilpivirine on cell-cycle distribution a flow cytometry-based analysis was performed after staining the untreated and treated cells with propidium iodide (PI) solution [22]. Briefly, 6 × 10^4^ cells/well were seeded in 3 mL of medium per well in 6-well plates. After overnight incubation at 37 °C in 5% CO_2_, the cells were incubated with the test compounds for specified time points. The medium-containing cells were then taken out from the wells and transferred to FACS tubes. Cells were then pelleted by centrifugation at 300 g for 5 min. Afterward, cells were fixed by using 70% (*v*/*v*) ice-cold ethanol for 15 min and then again pelleted by centrifugation (300 g for 5 min). Finally, the cells were incubated with 200 µL of PI staining solution (50 μg/mL PI, 0.1% sodium citrate, 0.1 mg/mL RNase A, and 0.1% Triton X-100) for 1.5 h at room temperature in the dark. After the incubation period, the cells were analysed using a CytoFLEX flow cytometer (Beckman Coulter Inc., Brea, CA, USA). CytExpert software version 2.1 was used for data analysis and graph preparation.

### 2.7. Apoptosis Analysis

The cellular apoptosis was detected using annexin-V/PI double staining by following a method described previously [22]. Briefly, cells were added in 6-well plates containing 3 mL of medium each well at 8 × 10^4^ cells/well and incubated at 37 °C, 5% CO_2_. After overnight incubation, the test compounds were added to the cells and again incubated for desired time points. Then, the cells were collected in FACS tubes, washed with PBS and incubated with annexin V and PI solution for 15 min in the dark at room temperature. The apoptotic cell death was measured within 1 h using the same instrument and data processing software described for cell cycle analysis.

### 2.8. Caspase-3/7 Activity Assay

Caspase-3/7 activity was measured after incubation of the cells with the test compounds for a specified time point using an Apo-ONE homogeneous caspase-3/7 kit (Promega, Madison, WI, USA) according to the manufacturer’s instructions. The signal was recorded by the EnVision multi-label plate reader (PerkinElmer, Beaconsfield, UK).

### 2.9. Western Blotting

Western blotting was performed following a method described previously [23]. Briefly, 8 × 10^5^ to 1 × 10^6^ cells were seeded in petri dishes with 8 to 10 mL of culture medium and incubated overnight at 37 °C, 5% CO_2_. Then, the cells were treated with the test compounds and further incubated for the specified time point. Cells were then lysed using a lysis buffer containing protease inhibitors, and proteins were extracted by centrifugation. Protein concentrations were determined by using a Bio-Rad DC^TM^ protein assay kit (Bio-Rad Laboratories, Hercules, CA, USA). A total of 20–30 μg of proteins was loaded onto each well of 4–20% polyacrylamide gels for electrophoresis. Once electrophoresis was completed, gels were placed on pre-soaked polyvinylidene difluoride membranes and blocked with 10% skimmed milk in 1 × Tris-buffered saline and Tween (TBST). Then, the blots were incubated with primary antibodies, followed by washing with TBST for 4 × 20 min. After this, the blots were incubated with the appropriate horseradish peroxidase (HRP)-conjugated secondary antibody, followed by washing again with TBST for 4 × 20 min. The protein bands were then detected with either ECL prime (Amersham^TM^ ECL^TM^ prime Western blotting detection reagent) or ECL select (Amersham^TM^ ECL^TM^ select Western blotting detection reagent) depending on the abundance of the protein of interest. The blots were imaged using the Bio-Rad ChemiDoc^TM^ MP imaging system (Bio-Rad Laboratories, Hercules, CA, USA). All the primary and secondary antibodies used in this study were purchased from Cell Signaling Technology (Danvers, MA, USA).

### 2.10. Animal Experiments

Animal experiments were performed as reported previously [24]. All animal experiments were conducted following the institutional ethical principles of animal care and approved by the University of South Australia Animal Ethics Committee.

### 2.11. Pharmacokinetic Analysis

A pharmacokinetic study was performed as described previously [24]. The pharmacokinetics of rilpivirine was determined after administration of single 50 mg/kg oral (PO) and 2 mg/kg intravenous (IV) doses to male BALB/c hairy mice (*n* = 6 per route). Briefly, three blood samples were collected from both cheeks (left and right) and the heart of each mouse. A validated liquid chromatography-mass spectrometry/mass spectrometry (LC-MS/MS) technique was used to measure the drug concentrations in the plasma. Pharmacokinetic parameters were estimated using compartmental models (Phoenix, Certara, NJ, USA) and analysis was performed with WinNonlin 6.4 software (Princeton, NJ, USA).

### 2.12. In Vivo Safety Profiles

Female 6–7-week-old BALB/c nude mice with tumour volumes of 100–200 mm^3^ were allocated to two groups (*n* = 3 per group) and treated with vehicle (0.5% HPMC in water) or rilpivirine (200 mg/kg) daily for 14 days. Upon 24 h post the final dose, mice were humanely killed, and blood, bone marrow, heart, kidney, intestine, liver, and stomach were collected for histopathological analysis. Blood was collected in citrate-treated tubes and a complete blood count was performed by Gribbles Veterinary Pathology (South Australia, Australia). Organs were collected in 10% neutral buffered formalin and stained with hematoxylin and eosin externally by the Hanson Histology Services (South Australia, Australia), while histopathologic analysis was performed by a board-certified pathologist.

### 2.13. In Vivo Anti-Cancer Efficacy Study

An in vivo anti-cancer efficacy study was performed as reported previously [23]. Briefly, female nude BALB/c mice (6–8 weeks of age) were engrafted with HL-60 human leukaemic cells subcutaneously into the rear flanks. Before inoculation, HL-60 (5 × 10^6^) cells were cultured, harvested, and re-suspended in a 1:1 mixture of serum-free media and Matrigel (BD Biosciences, NSW, Australia). Mice with 100–200 mm^3^ tumour size were allocated randomly into the control or treatment group. Rilpivirine and vehicle (0.5% HPMC) were administered by oral gavage daily. Mice were checked daily for any clinical signs of toxicity, including weight loss. Tumour volume was measured every other day and was calculated as length × width × height. At the end of the study period, or if clinical endpoints were reached as determined by a clinical record sheet score of 4, body condition score of 1, weight loss of ≥15%, or tumour growth of ≥2000 mm^3^, the mouse was euthanised. Data analysis was performed using GraphPad Prism software version 7.02 (La Jolla, CA, USA).

### 2.14. Statistical Analysis

All the statistical analyses were performed using GraphPad Prism software version 7.02 (La Jolla, CA, USA). Statistical significance between the two groups was determined using an unpaired Student’s *t*-test and, in the case of multiple groups, one-way analysis of variance (ANOVA) was used. The minimal level of significance was set at *p* < 0.05.

## 3. Results

### 3.1. Rilpivirine Shows Significant Inhibitory Selectivity towards Aurora A Kinase

**Figure 1:**
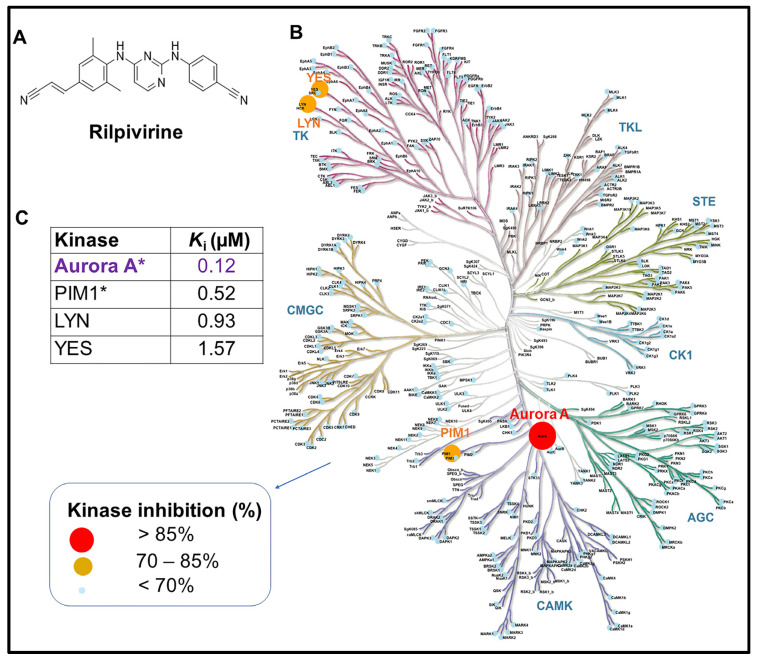
Structure and kinome-wide selectivity profile of rilpivirine. (**A**) Chemical structure of rilpivirine ((E)-4-((4-((4-(2-cyanoethenyl)-2,6-dimethylphenyl)amino)pyrimidin-2-yl)amino)benzonitrile). (**B**) Kinome map showing kinome-wide selectivity of rilpivirine at 1 μM concentration. The percentages of kinase inhibition mediated by rilpivirine are represented by coloured circles depicted in the legend. The kinase residual activities (%) remaining after treatment with rilpivirine of 429 tested kinases are included in Supplementary Table S1. (**C**) The apparent inhibition constant (*K*_i_) value of each kinase with >70% inhibitory activity is presented in the table. *K*_i_ value was calculated from the corresponding IC_50_ value and the appropriate *K*_m_ (ATP) value for each kinase. * Data were previously reported (in Ref [11]). The kinome map is reproduced by the courtesy of Cell Signalling Technology, Inc. Note: Atypical kinases tested were not depicted in the kinome map.

We previously reported that the diarylpyrimidine anti-viral drug rilpivirine (Figure 1A) inhibits Aurora A kinase potently with a *K*_i_ value of approximately 0.116 µM [11]. We also obtained a preliminary assessment of its kinase inhibitory activity against 48 well-established kinase targets at 10 µM [11]. In the present study, we sought to expand this investigation into the kinase inhibitory selectivity of this drug using a broader panel of kinases and at a lower assessment concentration.

Thus, rilpivirine was tested against a panel of 429 kinases representing >80% of the human protein kinases at the concentration of 1 μM. The results summarised in Figure 1B,C revealed that Aurora A was the most potently inhibited kinase by rilpivirine and this drug had no significant inhibitory activity against the vast majority of kinases tested except for only three other kinases, PIM1, LYN, and YES, where >70% inhibitory activity (i.e., ≤30% residual activity) was observed at 1 μM rilpivirine concentration. These data suggest that rilpivirine has appreciable selectivity for inhibiting Aurora A kinase in the human kinome (Figure 1 and Supplementary Table S1).

### 3.2. Rilpivirine Exhibits Anti-Proliferative Activities in Multiple Leukaemic Cell Lines

**Figure 2:**
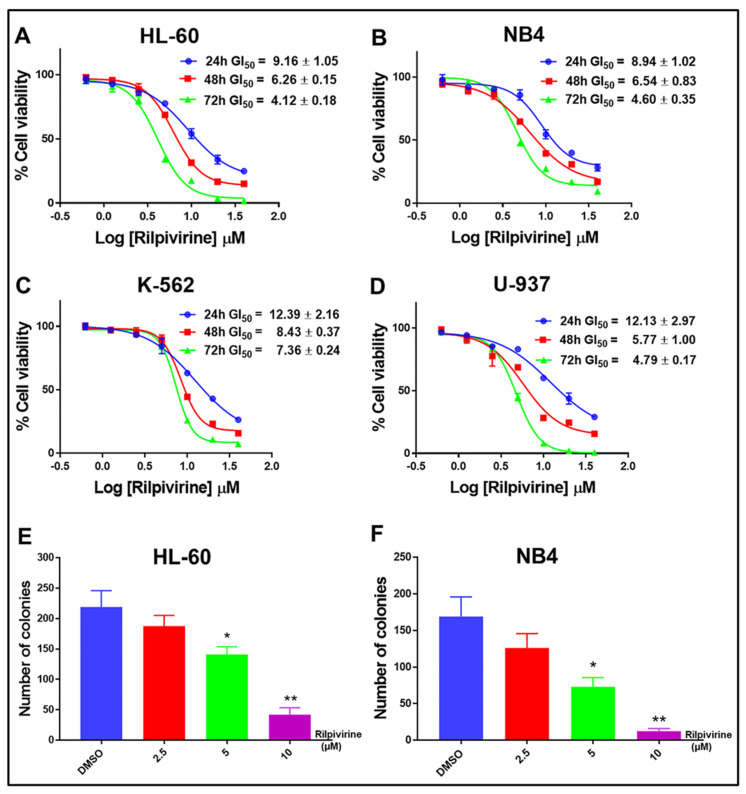
Growth inhibitory profile of rilpivirine against leukaemia cell lines. (**A**–**D**) Representative dose–response curves with GI_50_ values for HL-60, NB4, K-562, and U-937 cells following incubation with rilpivirine for the indicated times. Data (mean ± SD) were presented from at least three replicates. (**E**,**F**) The effect of rilpivirine on the clonogenicity of HL-60 and NB4 cells. Data (mean ± SD) were presented from at least two independent experiments (* *p* < 0.05, ** *p* < 0.01).

Rilpivirine was identified as a potential anti-cancer agent through ligand-based virtual screening and showed anti-cancer activities against a wide range of cancer cell lines, including leukaemia with single-digit micromolar concentrations [11]. Here, the time-dependent anti-proliferative activities of rilpivirine were assessed using resazurin cell viability assay in four leukaemia cell lines (HL-60, NB4, K-562, and U-937). These cell lines represent different genetic backgrounds and French–American–British (FAB) subtypes of AML [25] (Supplementary Table S2). Rilpivirine showed anti-proliferative activities against all the cell lines tested with GI_50_ values ranging from 4.12 to 12.39 μM after 24, 48, or 72 h of treatment, irrespective of their different characteristics (Figure 2A–D). Prolonged exposure to rilpivirine caused a greater reduction in the viability of these leukaemia cell lines.

These results suggested that rilpivirine displayed anti-proliferative activities against multiple leukaemia cell lines in both time- and concentration-dependent manners. Furthermore, rilpivirine treatment significantly decreased the clonogenic growth of HL-60 and NB4 cells in a concentration-dependent manner (Figure 2E,F and Supplementary Figure S1).

### 3.3. Rilpivirine Arrests Leukaemic Cells in the G_2_/M Phase of the Cell Cycle

**Figure 3:**
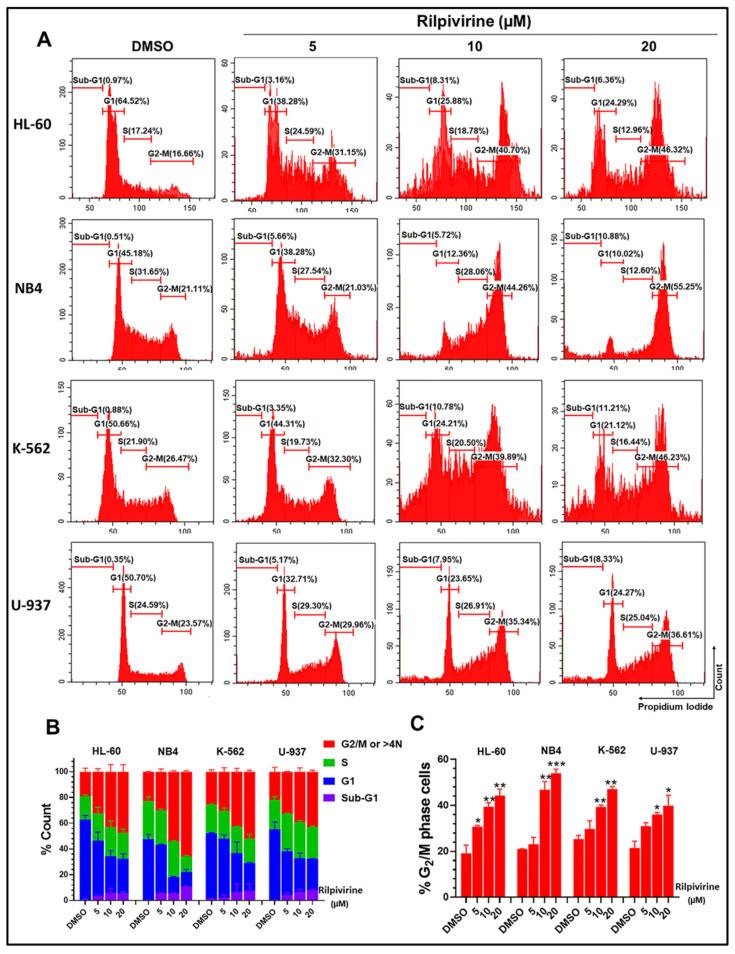
Cell-cycle analysis of leukaemic cells following treatment with rilpivirine. HL-60, NB4, K-562, and U-937 cells were incubated with 5, 10, or 20 μM rilpivirine for 24 h. (**A**) Representative histograms show the distribution of cells in different cell-cycle phases. (**B**) The percentages of cells distributed in different phases of the cell cycles of the tested cell lines. (**C**) The percentages of cells accumulated in the G_2_/M phases of the respective cell lines. Data (mean ± SD) were presented from at least two independent experiments (* *p* < 0.05, ** *p* < 0.01, and *** *p* < 0.001).

To investigate whether rilpivirine-mediated anti-proliferative activities in leukaemia cell lines were a consequence of cell-cycle arrest, the effects of rilpivirine on cell-cycle progression were examined by measuring cellular DNA content using flow cytometry. As shown in Figure 3, exposure of HL-60, NB4, K-562, and U-937 cells to 5, 10, or 20 µM rilpivirine for 24 h induced an accumulation of cells in the G_2_/M phase of the cell cycle in a concentration-dependent manner. For example, in the untreated (DMSO diluent-treated only) control, the majority of HL-60 cells (64.52%) accumulated in the G_1_ phase and only 16.66% of cells in the G_2_/M phase. However, when treated with rilpivirine for 24 h at concentrations of 5, 10, or 20 µM, 31.15%, 40.70%, and 46.32% of HL-60 cells were arrested in the G_2_/M phase, respectively.

Rilpivirine showed a similar influence on cell-cycle profiles with the other leukaemia cell lines tested characterised by an increased accumulation of cells in the G_2_/M phase and a reduction in the number of cells in the G_1_ phase at 5, 10, or 20 µM concentrations. Notably, the treatment of NB4, K-562, or U-937 cells with 20 µM rilpivirine significantly induced 55.25%, 46.23%, and 36.61% G_2_/M cells, while untreated cells displayed lower levels of G_2_/M cells (i.e., 21.11%, 26.47%, and 23.57%, respectively). It was observed that, at comparatively higher concentrations (10 and 20 µM), there was a slight accumulation of cells with >4N DNA content, indicative of endoreduplication.

Rilpivirine also displayed a time-dependent accumulation of HL-60 cells in the G_2_/M phase, with a maximum accumulation at 16 h. With prolonged exposure, a tendency for the cells to go into endoreduplication and sub-G_1_ phase was observed (Supplementary Figure S2).

### 3.4. Rilpivirine Induces Apoptosis and Caspase 3/7 Activation in Leukaemic Cells

**Figure 4:**
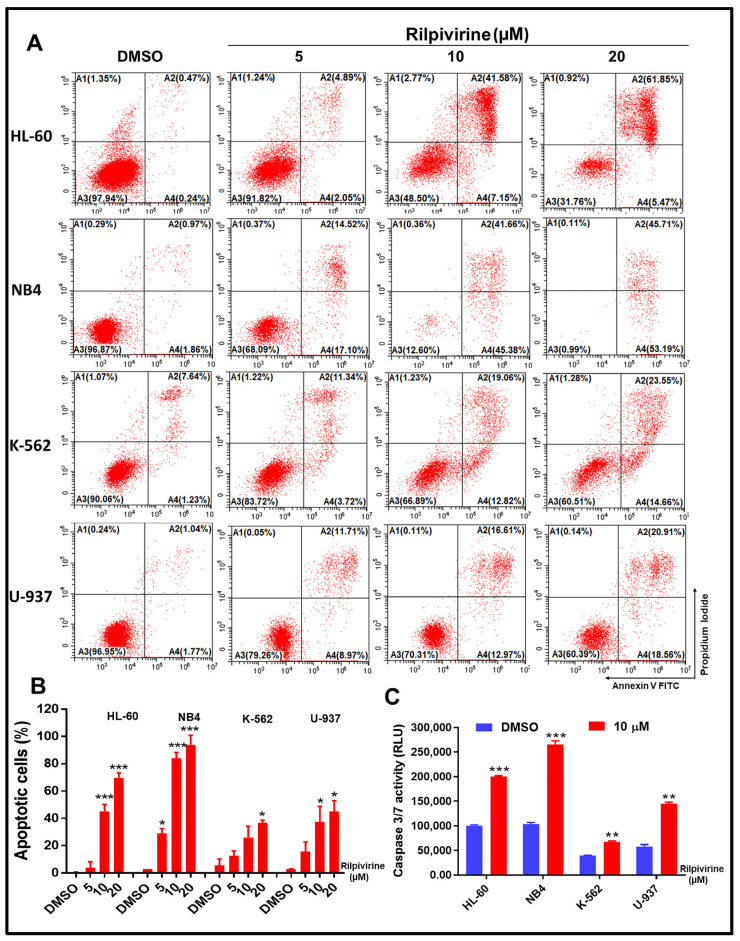
Effect of rilpivirine on apoptosis of leukaemic cells. HL-60, NB4, K-562, and U-937 cells were treated with rilpivirine for 48 h at the indicated concentrations and apoptosis was detected using annexin V/PI double staining. (**A**) Representative figures from flow cytometric analysis are shown. (**B**) The percentages of apoptotic cells at indicated concentrations. (**C**) The activity of caspase 3/7 in cells after incubation with 10 µM rilpivirine for 48 h. Data (mean ± SD) were presented from three independent experiments (* *p* < 0.05, ** *p* < 0.01, and *** *p* < 0.001).

Given the pronounced cell-cycle arrest caused by rilpivirine, we sought to quantify the induction of apoptosis by this drug through the annexin V/propidium iodide (PI) double staining. For this purpose, HL-60, NB4, K-562, and U-937 cells were incubated with rilpivirine at 5, 10, or 20 µM concentrations for 48 h. The results showed that rilpivirine induced significant apoptosis in a concentration-dependent manner (Figure 4A,B).

For example, 5 µM rilpivirine induced apoptosis (early and late apoptosis) in 6.94%, 31.62%, 15.06%, and 20.68% of HL-60, NB4, K-562, and U-937 cells, respectively. The apoptotic effects were enhanced with increasing rilpivirine concentrations in HL-60, NB4, K-562, and U-937 cells, inducing 48.73%, 87.04%, 31.88%, and 29.58% apoptotic cells at 10 µM rilpivirine and 67.32%, 98.9%, 38.21%, and 39.47% apoptotic cells at 20 µM rilpivirine, respectively (Figure 4A,B). When the four cell lines were exposed to the same concentrations of rilpivirine for 72 h, increased apoptotic responses were observed in all cell lines.

Apoptosis occurs as a result of the sequential activation of caspases, a family of cys proteases. Thus, to corroborate these results, we performed caspase 3/7 activity measurements in all cell lines using 10 µM rilpivirine for 48 h of exposure. The results were aligned with the annexin V/PI double staining results and indicated that caspase 3/7 activation was significantly increased following rilpivirine treatment compared with the untreated control (Figure 4C).

### 3.5. Rilpivirine Inhibits Aurora A Autophosphorylation and Modulates the Cell Cycle and Apoptosis Regulatory Proteins

**Figure 5:**
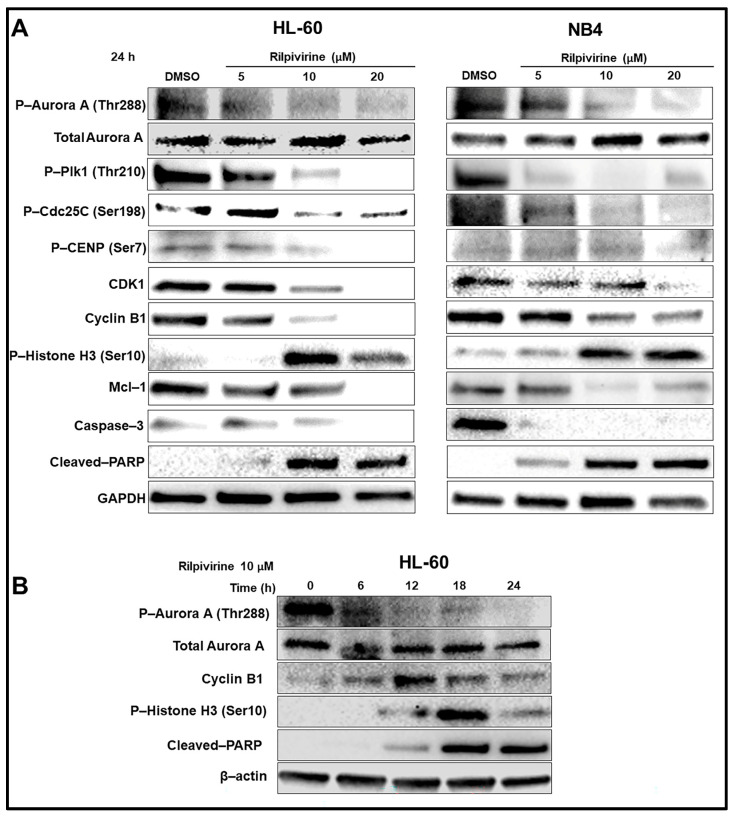
Mechanisms of anti-leukaemic action of rilpivirine. (**A**) Western blot analysis of HL-60 and NB4 cells treated with rilpivirine at indicated concentrations for 24 h. (**B**) Western blot analysis of HL-60 cells treated with 10 µM rilpivirine over different time intervals as indicated. Anti-bodies used are indicated on the left side of each panel, respectively. DMSO diluent is used as a control. GAPDH and β-actin are used as the internal loading controls. The results of at least two independent experiments are presented in the figure.

Western blot analysis was carried out to examine the cellular biological responses resulting from the rilpivirine-mediated inhibition of Aurora A kinase activity in leukaemic cells and the results are summarised in Figure 5A. The key proteins of the Aurora A kinase signalling pathway, including those known to be involved in the regulation of G_2_/M cell-cycle arrest and cellular apoptosis, were analysed. For this purpose, we selected two of the rilpivirine-sensitive cell lines, HL-60 and NB4. Exposure of HL-60 and NB4 cells to rilpivirine for 24 h suppressed the auto-phosphorylation of Aurora A at Thr288 in a concentration-dependent manner without affecting the total Aurora A, confirming the inhibition of Aurora A kinase activity by rilpivirine in both cell lines. It is now well-established that Plk1 activation is dependent on Aurora A kinase [26]. Plk1 phosphorylates the Cdc25C protein (phosphatase), which in turn activates the CDK1-cyclin B1 complex for facilitating mitotic entry [27,28]. Rilpivirine inhibited both Plk1 (Thr210) and Cdc25C (Ser198) phosphorylation, thus preventing the activation of the Aurora A-Plk1-Cdc25C cascade required for the onset of mitosis. Rilpivirine also reduced the CDK1 and cyclin B1 levels but induced the phospho-Histone H3 (Ser10), which is a marker for selective Aurora A kinase inhibition [29]. Moreover, rilpivirine inhibited the phosphorylation of CENP (Ser7) associated with kinetochore function during the prophase of mitosis [30]. There was also down-regulation of the anti-apoptotic protein Mcl-1 and increased PARP and caspase 3 cleavage, confirming apoptosis induction (Figure 5A).

HL-60 cells were further treated with rilpivirine over different time intervals. The inhibition of Aurora-A autophosphorylation was evident within 6 h of rilpivirine treatment (Figure 5B), implying Aurora-A kinase is a major target for rilpivirine action in the cellular context. In line with our previously described cell-cycle analysis results in HL-60 cells, cyclin B1 level was increased up to 12 h and then its level started to decrease. The induction of phospho-Histone H3 (Ser10) was maximum at 18 h (Figure 5B). PARP cleavage was also detected from 12 h and onwards (Figure 5B). Collectively, these results indicated an orchestrated time- and concentration-dependent expression of critical proteins associated with the inhibition of Aurora A kinase with rilpivirine in leukaemic cell lines.

### 3.6. Rilpivirine Inhibits Leukaemic Tumour Growth In Vivo

Next, the anti-leukaemic efficacy of rilpivirine was evaluated in vivo. Prior to examining the in vivo efficacy, we performed confirmative single-dose pharmacokinetic studies using BALB/c nude mice by an intravenous (IV) injection at 2 mg/kg or via oral gavage at 50 mg/kg (Supplementary Figure S3 and Supplementary Table S3). For both routes of administration, the maximum plasma concentration (C_max_) was above the effective concentration (>20 µM) required for modulating the expression of critical proteins related to proliferation. The tolerated dose of rilpivirine was confirmed using non-tumour-bearing female BALB/c nude mice (*n* = 3). Rilpivirine did not cause any significant changes in body weight (i.e., >15%) or show overt clinical signs of toxicity up to an oral dose of 300 mg/kg, once daily for seven days (Supplementary Figure S4). For the efficacy study, a dose of 200 mg/kg was used based on the above two preliminary experiments. A subcutaneous HL-60 xenograft murine model was used as described in the Materials and Methods section.

Initially, the safety profile of rilpivirine at 200 mg/kg dose was established. HL-60 leukaemic cells engrafted mice were treated with rilpivirine (200 mg/kg) or vehicle (i.e., 0.5% hydroxypropyl methylcellulose (HPMC) in water) orally, once daily for 14 consecutive days. At the end of the treatment, the mice were sacrificed and major organs including bone marrow, heart, kidney, intestine, liver, and stomach were collected for histopathological analysis. The analysis revealed that rilpivirine at 200 mg/kg dose did not cause any tissue damage in major organs (Supplementary Figure S5A). The complete blood count analysis following rilpivirine treatment did not show any noticeable effects on the red blood cells, neutrophils, lymphocytes, or platelets (Supplementary Figure S5B–E).

**Figure 6:**
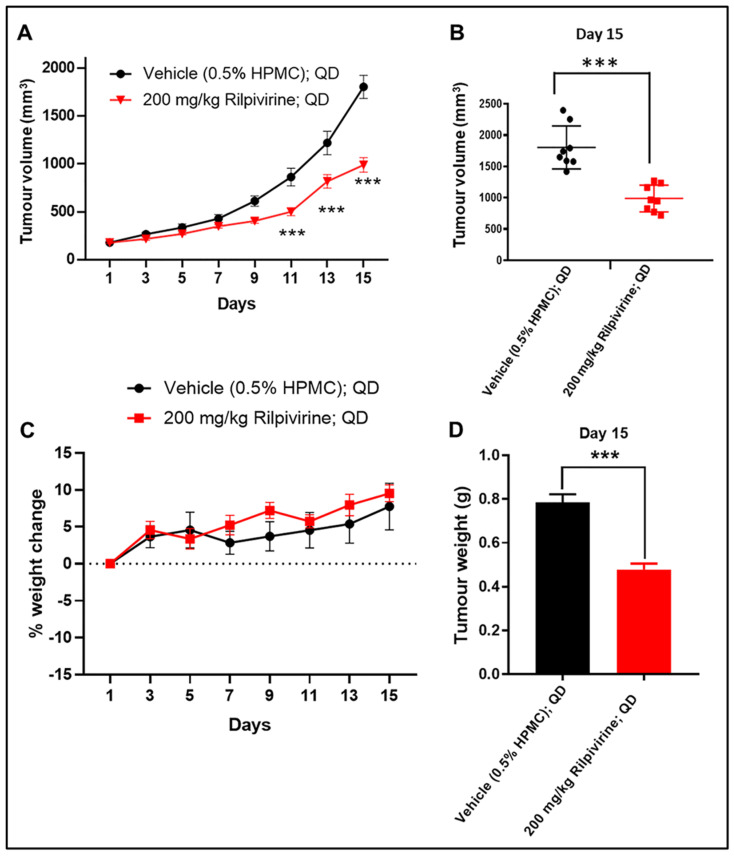
In vivo anti-leukaemic efficacy of rilpivirine in the HL-60 xenograft model. Animals (*n* = 8 per group) with subcutaneously implanted HL-60 leukaemia xenograft were treated with vehicle (0.5% HPMC in water, QD) or rilpivirine (200 mg/kg, QD) orally for 15 consecutive days. (**A**) Graph showing tumour volumes at different measurement days (*** *p* < 0.001; determined by one-way ANOVA). (**B**) Dot plot showing individual tumour volume on day 15 (horizontal line indicates the means, while vertical lines indicate standard deviations). *** *p* < 0.001; determined by unpaired Student’s *t*-test. (**C**) Body weight changes in different groups. Data presented as mean ± SEM. (**D**) Graph showing mean tumour weights of the vehicle and rilpivirine-treated groups on day 15. (*** *p* < 0.001; determined by unpaired Student’s *t*-test).

The in vivo anti-leukemic efficacy of rilpivirine was then determined. HL-60 leukaemic cells engrafted mice (*n* = 8) were fed with vehicle or 200 mg/kg rilpivirine via oral gavage once daily for 15 consecutive days. Mice were checked daily during dosing. Rilpivirine caused a noticeable delay in tumour growth compared with the untreated (vehicle-treated only) group (Figure 6A,B,D). A statistically significant reduction in tumour growth (*p* < 0.001) was observed from day 11 onwards compared with the vehicle-treated group (Figure 6A). Notably, throughout the course of treatment, rilpivirine did not cause any apparent signs of toxicity or weight loss (Figure 6C).

### 3.7. Rilpivirine Enhanced the Anti-Proliferative Effect of Cytarabine in Leukaemic Cells

**Figure 7:**
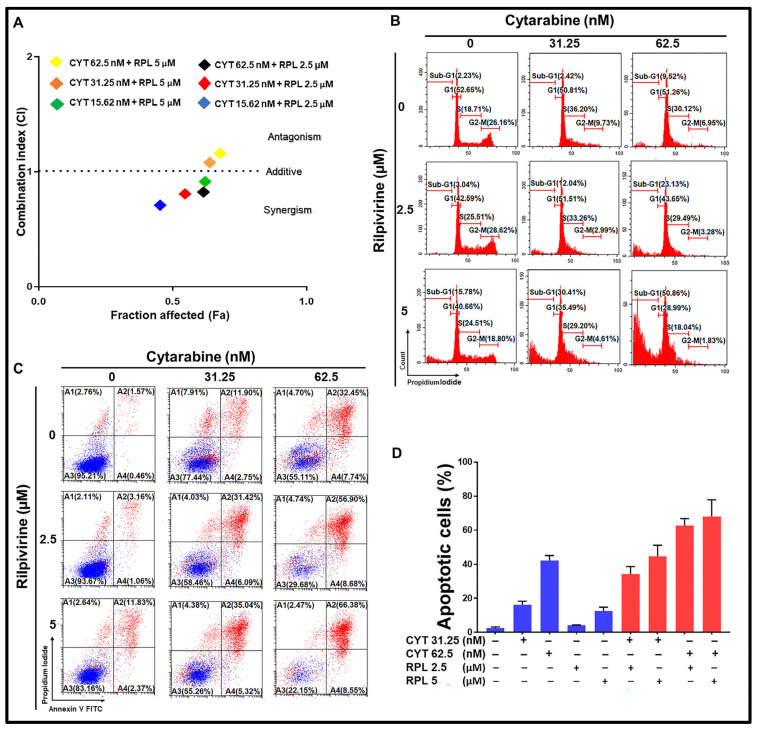
Effect of combination of rilpivirine and cytarabine in HL-60 cells. (**A**) Combination effects of rilpivirine and cytarabine at different concentrations using the 72-h resazurin assay and the combination indices (i.e., CI = 1 additive effect, CI < 1 synergy, or CI > 1 antagonism) are shown in a median effect plot (CYT, cytarabine and RPL, rilpivirine). The CI values were determined using the CompuSyn program (ComboSyn, Inc.). (**B**) Representative histograms of cell-cycle distribution are shown following treatment with indicated concentrations of rilpivirine and cytarabine either alone or in combination for 96 h. (**C**) Representative figures showing induction of apoptosis following treatment with indicated concentrations of rilpivirine and cytarabine either alone or in combination for 96 h and then measured using flow cytometry after annexin V-FITC/PI double staining. (**D**) The percentages of apoptotic cells following the same treatments are shown in the bar graph.

Rilpivirine was assessed to see if it could enhance the anti-proliferative effects of the conventional anti-leukaemic chemotherapy, cytarabine. First, the 72-h GI_50_ value of cytarabine was determined to be 70 nM in the HL-60 cell line. Then, HL-60 cells were treated with rilpivirine (at 2.5 or 5 µM) or cytarabine (at 15.62, 31.25, or 62.5 nM) as a single agent or in combination for 72 h and the cell viability was measured by the resazurin assay. The combination index (CI) values were calculated by using the median-effect analysis method [31,32]. The additive or slightly synergistic effects were observed when rilpivirine and cytarabine were used in combination at the lower doses (Figure 7A). Considering the Fa (fraction affected) and CI values, we next chose 2.5 and 5 µM for rilpivirine and 31.25 and 62.5 nM for cytarabine in the subsequent cell cycle and apoptosis analyses. The combination of rilpivirine and cytarabine induced higher apoptotic cell death (Figure 7C,D) compared with either single agent alone, which is consistent with an increased accumulation of sub-G1 cells (Figure 7B) and an increased PARP cleavage (Supplementary Figure S6). Collectively, these results indicated that rilpivirine can enhance the anti-proliferative effects of cytarabine.

## 4. Discussion

The current study explored the anti-leukaemic potential of the anti-viral drug rilpivirine attributed to its Aurora A kinase inhibitory role. In a recent study, we identified that rilpivirine is an Aurora A kinase inhibitor with low sub-micromolar potency [11]. Further in this study, we demonstrated with an extensive kinome assay that Aurora A was the most potently inhibited kinase by rilpivirine when it was tested against a panel of 429 kinases. This finding, rilpivirine, an aminopyrimidine, has Aurora A kinase inhibitory property, is entirely consistent with the findings of Wang et al., showing the Aurora A and Aurora B kinase inhibitory properties of a range of synthetic substituted pyrimidines [33]. Additional support for rilpivirine’s Aurora A kinase inhibition being the driver of the anti-leukaemic activity comes from subsequent experimentation using the leukaemia cell lines. A key feature of the findings is that the treatment of leukaemic cells with rilpivirine displayed the key hallmarks of Aurora A kinase inhibition, namely G_2_/M cell-cycle arrest, induction of phospho-Histone H3 (Ser10), and suppression of the auto-phosphorylation of Aurora A at Thr288 [4,34]. Furthermore, the other key proteins of the Aurora A kinase-signalling pathway were influenced by rilpivirine in a fashion entirely consistent with the inhibition of Aurora A kinase. These included rilpivirine-mediated inhibition of the phosphorylation of Plk1 (Thr210), Cdc25C (Ser 198), and CENP (Ser7) [26,28,30].

It is noteworthy that in the kinome-wide profiling of rilpivirine (at 1 µM concentration against 429 kinases) only three kinases—PIM1, LYN, and YES—displayed notable inhibition (>70%) by this compound, apart from Aurora A kinase (Figure 1). PIM1 was the nearest kinase of Aurora A in displaying an inhibitory potential of rilpivirine and this was four-and-a-half-fold less potent than Aurora A kinase. We believe the possibility that rilpivirine may exert its cellular inhibitory action through PIM1 kinase is very low. This is based on the observation that the expression of PIM1 kinase could not be detected in three AML cell lines examined (HL-60, NB4, and U937) (Supplementary Figure S7), consistent with previous reports [35,36]. Notably, one chronic myeloid leukaemia cell line, K-562, was included in some preliminary experiments of this study, considering its higher expression of PIM1 [37]. Consistently, the K-562 cell line showed overexpression of PIM1 (Supplementary Figure S7), but surprisingly it was not sensitive to the selective and potent PIM1 kinase inhibitor, AZD1208 (*K*_i_ against PIM1 < 1 nM), and displayed comparatively high GI_50_ value (~38 µM) (Supplementary Figure S8A). Importantly, AZD1208 induced G_1_ arrest and not G_2_/M cell-cycle arrest characteristic of Aurora A kinase inhibition in the K-562 cell line (Supplementary Figure S8B). It is worth mentioning that a previous study reported the structural and topological similarity of Aurora A with YES and LYN kinases [38]. However, we are unaware of any references reporting these two kinases in mitotic regulation such as Aurora A kinase. Notably, YES and LYN kinases are both members of the Src family kinases (SFKs) and are implicated in cancer [39,40]. In particular, LYN kinase has been shown to be over-expressed in AML cells [40,41]. Similar to PIM1 kinase, the inhibition of LYN kinase by rilpivirine is unlikely to mediate its anti-leukaemic activity in the context of the concentrations of rilpivirine used in this study. This conclusion is based on the comparison of the cell-cycle dynamics of KG1 cells following treatment with rilpivirine and an SFK inhibitor, PP2. A previous study reported that the SFK inhibitor PP2 induced G_1_ cell-cycle arrest in the KG1 cell line [40]. In contrast, rilpivirine induced G_2_/M cell-cycle arrest, a characteristic phenotype of Aurora A kinase inhibition (Supplementary Figure S9). There also exists a considerable concentration differential between the rilpivirine-mediated inhibition of YES or LYN kinases and that of Aurora A kinase. However, we cannot rule out with certainty some contribution of any of those kinases to rilpivirine’s inhibitory action on AML cell lines. Therefore, this pleiotropic kinase inhibitory role of rilpivirine should be explored further.

In this study, we showed that rilpivirine displayed almost similar time- and concentration-dependent anti-proliferative activity in HL-60, NB4, K-562, and U-937 leukaemia cell lines (Figure 2A–D). This correlates with the similar Aurora A expression levels in HL-60, NB4, and U-937 cell lines and a slightly lower expression level of Aurora A in the K-562 cell line, which we reported previously [11]. In addition, as the cells cannot continue to proliferate in the presence of rilpivirine, the clonal growth was also inhibited, as evident in the HL-60 and NB4 cell lines (Figure 2E,F). We also established that rilpivirine induced cell-cycle arrest at the G_2_/M phase in all the cell lines in a similar fashion to the selective Aurora A kinase inhibitor alisertib [42]. In eukaryotic cells, activation of the CDK1/cyclin B1 complex, the “mitosis-promoting factor” triggers the initiation of mitosis [43,44]. Cdc25C is phosphorylated by Plk1 and phosphorylated Cdc25C catalyses CDK1 into its unphosphorylated state or active form and thus regulates CDK1/cyclin B1 activity and controls cell-cycle progression from G_2_ to mitosis [45,46]. Rilpivirine inhibited Aurora A kinase activity by inhibiting the autophosphorylation of Aurora A at Thr288 and subsequently inhibited its downstream target Plk1 activation. The G_2_/M cell-cycle arrest observed in all the cell lines is potentially related to the disruption of the Aurora A-Plk1-Cdc25C interactions. Furthermore, after treatment with higher concentrations of rilpivirine, cells tend to undergo endoreduplication. This may be due to the absence of functional p53-mediated post-mitotic checkpoints in these cell lines [47]. Of importance was the observation that the structurally similar reverse transcriptase inhibitor etravirine failed to induce a G_2_/M phase arrest (Supplementary Figure S10), consistent with its inability to inhibit Aurora A kinase [11], further evidence for the selective action of rilpivirine.

Rilpivirine also showed time- and concentration-dependent apoptosis in all the leukaemia cell lines examined. Since cells cannot be arrested in mitosis indefinitely, they are forced to activate the apoptotic process [48]. It is well established that the balance between the pro-apoptotic and anti-apoptotic B-cell lymphoma-2 (BCL-2) family members at the mitochondrial membrane determines the death or survival of the cell [49]. As shown in Figure 5, expression levels of Mcl-1, one of the Bcl-2 family members, were significantly reduced in rilpivirine-treated HL-60 and NB4 cells. Cleavage of PARP has been used extensively as a marker of cells undergoing apoptosis [50,51]. Rilpivirine induced dose-dependent PARP cleavage, again confirming apoptosis induction. Moreover, the activation of caspase 3/7 also indicated the initiation of a caspase-dependent apoptotic pathway in these cell lines. Given that the FLT3-ITD mutation is one of the most common mutations in AML and all the previously tested cell lines were a FLT3 wild type, we extended our observations regarding the rilpivirine-mediated apoptotic response using the MV4-11 cell line, which has FLT3-ITD mutation. Rilpivirine induced significant apoptosis in MV4-11 cell lines (Supplementary Figure S11), suggesting its ability to induce apoptosis irrespective of the FLT3 status of AML cells.

In the present study, we explored the potential anti-cancer effects of rilpivirine using leukaemia cell lines for two key reasons. Firstly, Aurora kinase inhibitors appear to have greater potential with haematologic malignancies than with solid tumours, possibly due to a slower proliferation rate in the solid tumours [52]. Secondly, the overexpression of Aurora kinases as well as the high homogeneity in haematologic malignancies suggests potential therapeutic responsiveness to Aurora A kinase inhibition [52]. Accordingly, if the inhibitory influence of rilpivirine upon Aurora A kinase is functionally linked to the induction of cancer cell death, it should be evident in leukaemia cell lines. The results of our present study provide substantial evidence for a rilpivirine-mediated inhibition of proliferation and enhanced cell death of leukaemia cells. Importantly, rilpivirine suppresses tumour growth in mouse explants of HL-60 leukaemia cells. Of interest is the potential of rilpivirine to enhance the efficacy of established chemotherapeutic agents for AML treatment. Kelly et al. have drawn attention to the role of the selective Aurora A kinase inhibitor alisertib in increasing the efficacy of cytarabine in a forkhead box class O (FOXO)-dependent manner in AML [6]. Similarly, we also observed that rilpivirine could enhance the anti-proliferative and pro-apoptotic effects of the leading anti-leukaemic chemotherapeutic, cytarabine. We also demonstrated that the combination of rilpivirine with cytarabine caused a profound reduction in FOXO-3a phosphorylation and an increase in PARP cleavage compared with the use of either single agent alone in HL-60 cells (Supplementary Figure S6). Collectively, these findings suggest the role of rilpivirine-mediated inhibition of Aurora A kinase either solely or in combination with other properties of rilpivirine in inhibiting the proliferation of leukaemic cells. Moreover, the dose-limiting toxicities, including neutropenia, somnolence, and mucositis, may impede the development of specifically-synthesised Aurora A kinase inhibitors [53]. One advantage in the potential repurposing of rilpivirine as an Aurora A kinase inhibitor, assuming clinical efficacy, is the fact that it has been an established drug used in society for many years.

It is increasingly evident that anti-viral drugs, specifically the non-nucleoside reverse transcriptase inhibitors (NNRTIs) developed to treat HIV infection, display pharmacological pleiotropy and as such, in addition to their anti-viral action, can also inhibit cancer-cell proliferation. In a comparison of six different NNRTIs, cytotoxicity was demonstrated for all six compounds tested across a broad range of concentrations in cancer cells [54]. As highlighted by Fattore et al., nucleoside and non-nucleoside reverse transcriptase inhibitors have been shown to display anti-cancer properties in selected carcinoma cell lines [55]. By explanatory summary, Hecht et al. drew upon reports characterising the role of NNRTIs in the inhibition of endogenous reverse transcriptase, interaction with the cannabinoid system, and oxidative mitochondrial stress [54]. In a key study by Perna et al., a series of reverse transcriptase inhibitors (i.e., abacavir, tenofovir, efavirenz, etravirine, and darunavir) was tested with SKOV-3 ovarian-cancer cells and all these inhibitors resulted in a predominance of a G_0_/G_1_ phase arrest, as measured by fluorescence-activated cell sorting (FACS) analysis [56]. As indicated by Hecht et al., efavirenz treatment reduces the expression of cyclin D1 consistent with G_0_/G_1_ cell-cycle arrest [54]. Of importance is the observation by Perna et al., where treatment of SKOV-3 cells with etravirine, a molecule with high structural similarity to rilpivirine, failed to increase the proportion of cells in the G_2_/M phase as assessed by FACS analysis [56]. When contrasted with our present findings showing rilpivirine-induced G_2_/M arrest in leukaemia cells, it suggests a high specificity for the interwoven processes of Aurora A kinase inhibition and G_2_/M cell-cycle arrest.

It is noteworthy that historically the application of highly active anti-retroviral therapy (HAART) drew attention to their potential anti-tumour properties in view of their success in treating HIV-related Kaposi’s sarcoma [57]. Kaposi’s sarcoma is a common malignancy associated with acquired immunodeficiency syndrome (AIDS) believed to involve Kaposi’s sarcoma herpesvirus (KSHV) or human herpesvirus type 8 (HHV-8) [58]. Of significance to that study is the observation that KSHV-encoded latency-associated nuclear antigen (LANA) upregulates the transcriptional expression of Aurora A and this upregulated expression is markedly elevated in Kaposi’s sarcoma tissue and human primary cells infected with KSHV [59]. Additionally, the role of the oncovirus antigen LANA in cleaving Aurora B to induce host cell tumourigenesis has been suggested [60]. A very recent study also implicated Aurora A kinase in the HIV infection process [61]. Collectively, these observations provide the stimulus to explore further the role of rilpivirine and related molecules on the interaction between these oncoviruses and Aurora kinases.

A key question relates to whether the blood concentrations of rilpivirine (for example, the C_max_) for its approved use as an anti-viral drug in HIV are sufficient to inhibit the proliferation of leukaemia cells. Hecht et al. detailed the drug concentrations achieved in patients using NNRTIs including rilpivirine and compared them with the concentrations needed to display cytotoxicity in pancreatic cancer cells [54]. Based on their considerations [54] and our calculations, the effective anti-cancer concentration of rilpivirine would be higher than that normally seen in patients taking this drug as a therapy for HIV infection. Indeed, the requirement of higher doses is potentially an issue for many repurposing candidates in cancer that demands dose-finding trials with safety assessment [62]. For example, many repurposing candidates including fluvastatin, omeprazole, propranolol, and candesartan have been pre-clinically evaluated at higher doses for cancer indications and high doses (8 mg/kg) of fluvastatin were administered safely in children [62]. Regardless, this argues for additional studies using lower doses of rilpivirine, particularly in combination with established chemotherapeutic agents or targeted therapies. It will also be important to conduct the in vivo studies using different dose levels and dosing frequencies and explore the pharmacokinetic–pharmacodynamic relationships. It is important to note that the leukaemia cell lines used in this study were all p53 null or mutant cell lines. Considering the critical interactions of Aurora A kinase with p53 [63], it will be important to expand these studies with rilpivirine using cell lines with functional p53. Another limitation of this study is the absence of experiments examining the effect of rilpivirine on primary AML cells and, as such, the current findings should be viewed as proof of concept only, awaiting further investigation on primary cells in the future. Moreover, the translation of anti-leukemic activity of rilpivirine in patients might be challenging as many drug candidates with promising efficacy in cancer cell lines and animal-based studies often fail to maintain the same results in the clinic. Therefore, caution must be exercised regarding the findings in this study in the context of AML patients and additional pre-clinical validation should be conducted.

## 5. Conclusions

In the current study, we showed that rilpivirine, originally developed as an HIV anti-viral drug, exhibited significant anti-leukaemic activity. We established, for the first time, the selectivity of rilpivirine for Aurora A kinase inhibition in a panel of kinases, nearly reflecting the whole human kinome. Consistent with its Aurora A kinase inhibitory role, rilpivirine effectively inhibited leukaemic cell growth by inducing G_2_/M cell-cycle arrest and apoptosis and modulated the expression of critical proteins in the Aurora A kinase-signalling pathway. Oral rilpivirine treatment significantly inhibited leukaemic tumour growth in vivo. In addition, rilpivirine possessed the ability to enhance the anti-proliferative activity of the leading anti-leukaemia chemotherapy, cytarabine. Overall, this study provides a rationale for the further evaluation of rilpivirine to explore its full anti-leukaemic potential in the context of potential repurposing.

## Data Availability

Data presented in this study are available on request from the corresponding author.

## Supplementary Materials

The following supporting information can be downloaded at: https://www.mdpi.com/article/10.3390/cancers15041044/s1, Table S1. Kinome-wide selectivity of rilpivirine at 1 µM; Table S2. Genetic backgrounds and French–American–British (FAB) subtypes of tested cell lines; Figure S1. Effect of rilpivirine on the formation of colonies of HL-60 and NB4 cells; Figure S2. Cell cycle analysis of HL-60 cells following rilpivirine treatment (10 µM) at indicated time points; Figure S3. Plasma concentration-time profile of rilpivirine in mice after a single oral dose of 50 mg/kg (A) or a single IV dose of 2 mg/kg (B); Table S3. Pharmacokinetic properties of rilpivirine in mice; Figure S4. Effect of rilpivirine on body weight of mice; Figure S5. In vivo toxicity assessment of rilpivirine in mice; Figure S6. Western blot analysis of HL-60 cells treated with rilpivirine, cytarabine, or the combination at indicated concentrations for 72 h; Figure S7. PIM1 expression across a panel of myeloid leukaemia cell lines shown by Western blot analysis of cell lysates; Figure S8. (A) Dose–response curve for AZD1208 against K-562 cells at 72 h. (B) Cell cycle analysis of K-562 cells after incubation with AZD1208 (60 μM) for 48 h; Figure S9. Cell cycle analysis of KG1 cells following treatment with 10 µM or 20 µM rilpivirine for 24 h; Figure S10. Cell cycle analysis of NB4, U-937, and K-562 cells following treatment with 10 µM etravirine for 24 h; Figure S11. Effect of rilpivirine on apoptosis of MV4-11 cells; Figure S12. Original images of Western blots for Figure 5A, left panel (HL-60); Figure S13. Original images of Western blots for Figure 5A, left panel (HL-60) (continued); Figure S14. Original images of Western blots for Figure 5A, right panel (NB4); Figure S15. Original images of Western blots for Figure 5A, right panel (NB4) (continued); Figure S16. Original images of Western blots for Figure 5B (HL-60 time course); Figure S17. Original images of Western blots for Supplementary Figure S6; Figure S18. Original images of Western blots for Supplementary Figure S7.

## References

[1] Khwaja A., Bjorkholm M., Gale R.E., Levine R.L., Jordan C.T., Ehninger G., Bloomfield C.D., Estey E., Burnett A., Cornelissen J.J. (2016). Acute myeloid leukaemia. Nat. Rev. Dis. Prim..

[2] Döhner H., Weisdorf D.J., Bloomfield C.D. (2015). Acute myeloid leukemia. N. Engl. J. Med..

[3] DiNardo C.D., Jonas B.A., Pullarkat V., Thirman M.J., Garcia J.S., Wei A.H., Konopleva M., Döhner H., Letai A., Fenaux P. (2020). Azacitidine and venetoclax in previously untreated acute myeloid leukemia. N. Engl. J. Med..

[4] Huang X.-F., Luo S.-K., Xu J., Li J., Xu D.-R., Wang L.-H., Yan M., Wang X.-R., Wan X.-B., Zheng F.-M. (2008). Aurora kinase inhibitory vx-680 increases bax/bcl-2 ratio and induces apoptosis in aurora-a-high acute myeloid leukemia. Blood J. Am. Soc. Hematol..

[5] Crossnohere N.L., Richardson D.R., Reinhart C., O’Donoghue B., Love S.M., Smith B.D., Bridges J.F. (2019). Side effects from acute myeloid leukemia treatment: Results from a national survey. Curr. Med. Res. Opin..

[6] Kelly K.R., Nawrocki S.T., Espitia C.M., Zhang M., Yang J.J., Padmanabhan S., Ecsedy J., Giles F.J., Carew J.S. (2012). Targeting aurora a kinase activity with the investigational agent alisertib increases the efficacy of cytarabine through a foxo-dependent mechanism. Int. J. Cancer.

[7] Perl A.E. (2017). The role of targeted therapy in the management of patients with aml. Blood Adv..

[8] Stegmeier F., Warmuth M., Sellers W., Dorsch M. (2010). Targeted cancer therapies in the twenty-first century: Lessons from imatinib. Clin. Pharmacol. Ther..

[9] Islam S., Wang S., Bowden N., Martin J., Head R. (2022). Repurposing existing therapeutics, its importance in oncology drug development: Kinases as a potential target. Br. J. Clin. Pharmacol..

[10] Knapp S. (2018). New Opportunities for Kinase Drug Repurposing and Target Discovery.

[11] Islam S., Teo T., Kumarasiri M., Slater M., Martin J.H., Wang S., Head R. (2022). Combined in silico and in vitro evidence supporting an aurora a kinase inhibitory role of the anti-viral drug rilpivirine and an anti-proliferative influence on cancer cells. Pharmaceuticals.

[12] Malumbres M., Perez de Castro I. (2014). Aurora kinase a inhibitors: Promising agents in antitumoral therapy. Expert Opin. Ther. Targets.

[13] Mou P.K., Yang E.J., Shi C., Ren G., Tao S., Shim J.S. (2021). Aurora kinase a, a synthetic lethal target for precision cancer medicine. Exp. Mol. Med..

[14] Yan M., Wang C., He B., Yang M., Tong M., Long Z., Liu B., Peng F., Xu L., Zhang Y. (2016). Aurora-a kinase: A potent oncogene and target for cancer therapy. Med. Res. Rev..

[15] Goldenson B., Crispino J.D. (2015). The aurora kinases in cell cycle and leukemia. Oncogene.

[16] Kim S.-J., Jang J.E., Cheong J.-W., Eom J.-I., Jeung H.-K., Kim Y., Hwang D.Y., Min Y.H. (2012). Aurora a kinase expression is increased in leukemia stem cells, and a selective aurora a kinase inhibitor enhances ara-c-induced apoptosis in acute myeloid leukemia stem cells. Korean J. Hematol..

[17] Ikezoe T., Yang J., Nishioka C., Tasaka T., Taniguchi A., Kuwayama Y., Komatsu N., Bandobashi K., Togitani K., Koeffler H.P. (2007). A novel treatment strategy targeting aurora kinases in acute myelogenous leukemia. Mol. Cancer Ther..

[18] Fathi A.T., Wander S.A., Blonquist T.M., Brunner A.M., Amrein P.C., Supko J., Hermance N.M., Manning A.L., Sadrzadeh H., Ballen K.K. (2017). Phase i study of the aurora a kinase inhibitor alisertib with induction chemotherapy in patients with acute myeloid leukemia. Haematologica.

[19] Brunner A.M., Blonquist T.M., DeAngelo D.J., McMasters M., Winer E.S., Hobbs G.S., Amrein P.C., Hock H., Steensma D.P., Garcia J.S. (2018). Phase ii clinical trial of alisertib, an aurora a kinase inhibitor, in combination with induction chemotherapy in high-risk, untreated patients with acute myeloid leukemia. Blood.

[20] Farag S.S. (2011). The potential role of aurora kinase inhibitors in haematological malignancies. Br. J. Haematol..

[21] Cheng Y.-C. (1973). Relationship between the inhibition constant (ki) and the concentration of inhibition, which causes 50% inhibition (I50) of an enzymatic reaction. Biochem. Pharmacol..

[22] Diab S., Abdelaziz A.M., Li P., Teo T., Basnet S.K., Noll B., Rahaman M.H., Lu J., Hou J., Yu M. (2017). Dual inhibition of mnk2 and flt3 for potential treatment of acute myeloid leukaemia. Eur. J. Med. Chem..

[23] Rahaman M.H., Yu Y., Zhong L., Adams J., Lam F., Li P., Noll B., Milne R., Peng J., Wang S. (2019). Cdki-73: An orally bioavailable and highly efficacious cdk9 inhibitor against acute myeloid leukemia. Investig. New Drugs.

[24] Anshabo A.T., Bantie L., Diab S., Lenjisa J., Kebede A., Long Y., Heinemann G., Karanjia J., Noll B., Basnet S.K. (2022). An orally bioavailable and highly efficacious inhibitor of cdk9/flt3 for the treatment of acute myeloid leukemia. Cancers.

[25] Jensen H.A., Yourish H.B., Bunaciu R.P., Varner J.D., Yen A. (2015). Induced myelomonocytic differentiation in leukemia cells is accompanied by noncanonical transcription factor expression. FEBS Open Biol..

[26] Macůrek L., Lindqvist A., Lim D., Lampson M.A., Klompmaker R., Freire R., Clouin C., Taylor S.S., Yaffe M.B., Medema R.H. (2008). Polo-like kinase-1 is activated by aurora a to promote checkpoint recovery. Nature.

[27] Gheghiani L., Loew D., Lombard B., Mansfeld J., Gavet O. (2017). Plk1 activation in late g2 sets up commitment to mitosis. Cell Rep..

[28] Liu K., Zheng M., Lu R., Du J., Zhao Q., Li Z., Li Y., Zhang S. (2020). The role of cdc25c in cell cycle regulation and clinical cancer therapy: A systematic review. Cancer Cell Int..

[29] Shimomura T., Hasako S., Nakatsuru Y., Mita T., Ichikawa K., Kodera T., Sakai T., Nambu T., Miyamoto M., Takahashi I. (2010). Mk-5108, a highly selective aurora-a kinase inhibitor, shows antitumor activity alone and in combination with docetaxel. Mol. Cancer Ther..

[30] Kunitoku N., Sasayama T., Marumoto T., Zhang D., Honda S., Kobayashi O., Hatakeyama K., Ushio Y., Saya H., Hirota T. (2003). Cenp-a phosphorylation by aurora-a in prophase is required for enrichment of aurora-b at inner centromeres and for kinetochore function. Dev. Cell.

[31] Chou T.-C. (2006). Theoretical basis, experimental design, and computerized simulation of synergism and antagonism in drug combination studies. Pharmacol. Rev..

[32] Chou T.-C., Talalay P. (1984). Quantitative analysis of dose-effect relationships: The combined effects of multiple drugs or enzyme inhibitors. Adv. Enzym. Regul..

[33] Wang S., Midgley C.A., Scaërou F., Grabarek J.B., Griffiths G., Jackson W., Kontopidis G., McClue S.J., McInnes C., Meades C. (2010). Discovery of n-phenyl-4-(thiazol-5-yl) pyrimidin-2-amine aurora kinase inhibitors. J. Med. Chem..

[34] Wen Q., Goldenson B., Silver S.J., Schenone M., Dancik V., Huang Z., Wang L.-Z., Lewis T.A., An W.F., Li X. (2012). Identification of regulators of polyploidization presents therapeutic targets for treatment of amkl. Cell.

[35] Keeton E.K., McEachern K., Dillman K.S., Palakurthi S., Cao Y., Grondine M.R., Kaur S., Wang S., Chen Y., Wu A. (2014). Azd1208, a potent and selective pan-pim kinase inhibitor, demonstrates efficacy in preclinical models of acute myeloid leukemia. Blood J. Am. Soc. Hematol..

[36] Cen B., Xiong Y., Song J.H., Mahajan S., DuPont R., McEachern K., DeAngelo D.J., Cortes J.E., Minden M.D., Ebens A. (2014). The pim-1 protein kinase is an important regulator of met receptor tyrosine kinase levels and signaling. Mol. Cell. Biol..

[37] Alsubaie M., Matou-Nasri S., Aljedai A., Alaskar A., Al-Eidi H., Albabtain S.A., Aldilaijan K.E., Alsayegh M., Alabdulkareem I.B. (2021). In vitro assessment of the efficiency of the pim-1 kinase pharmacological inhibitor as a potential treatment for burkitt’s lymphoma. Oncol. Lett..

[38] Cheetham G.M., Knegtel R.M., Coll J.T., Renwick S.B., Swenson L., Weber P., Lippke J.A., Austen D.A. (2002). Crystal structure of aurora-2, an oncogenic serine/threonine kinase. J. Biol. Chem..

[39] Garmendia I., Redin E., Montuenga L.M., Calvo A. (2022). Yes1: A novel therapeutic target and biomarker in cancer. Mol. Cancer Ther..

[40] Dos Santos C., Demur C., Bardet V., Prade-Houdellier N., Payrastre B., Récher C. (2008). A critical role for lyn in acute myeloid leukemia. Blood J. Am. Soc. Hematol..

[41] Patel R.K., Weir M.C., Shen K., Snyder D., Cooper V.S., Smithgall T.E. (2019). Expression of myeloid src-family kinases is associated with poor prognosis in aml and influences flt3-itd kinase inhibitor acquired resistance. PLoS ONE.

[42] Liu Z., Wang F., Zhou Z.-W., Xia H.-C., Wang X.-Y., Yang Y.-X., He Z.-X., Sun T., Zhou S.-F. (2017). Alisertib induces g_2_/m arrest, apoptosis, and autophagy via pi3k/akt/mtor-and p38 mapk-mediated pathways in human glioblastoma cells. Am. J. Transl. Res..

[43] Jackman M., Lindon C., Nigg E.A., Pines J. (2003). Active cyclin b1–cdk1 first appears on centrosomes in prophase. Nat. Cell Biol..

[44] Nurse P. (1990). Universal control mechanism regulating onset of m-phase. Nature.

[45] Toyoshima-Morimoto F., Taniguchi E., Nishida E. (2002). Plk1 promotes nuclear translocation of human cdc25c during prophase. EMBO Rep..

[46] Schmit T.L., Ahmad N. (2007). Regulation of mitosis via mitotic kinases: New opportunities for cancer management. Mol. Cancer Ther..

[47] Kojima K., Konopleva M., Tsao T., Nakakuma H., Andreeff M. (2008). Concomitant inhibition of mdm2-p53 interaction and aurora kinases activates the p53-dependent postmitotic checkpoints and synergistically induces p53-mediated mitochondrial apoptosis along with reduced endoreduplication in acute myelogenous leukemia. Blood J. Am. Soc. Hematol..

[48] Oussenko I.A., Holland J.F., Reddy E.P., Ohnuma T. (2011). Effect of on 01910. Na, an anticancer mitotic inhibitor, on cell-cycle progression correlates with rangap1 hyperphosphorylation. Cancer Res..

[49] Brenner D., Mak T.W. (2009). Mitochondrial cell death effectors. Curr. Opin. Cell Biol..

[50] Oliver F.J., de la Rubia G., Rolli V., Ruiz-Ruiz M.C., de Murcia G., Ménissier-de Murcia J. (1998). Importance of poly (adp-ribose) polymerase and its cleavage in apoptosis lesson from an uncleavable mutant. J. Biol. Chem..

[51] Soldani C., Lazzè M.C., Bottone M.G., Tognon G., Biggiogera M., Pellicciari C.E., Scovassi A.I. (2001). Poly (adp-ribose) polymerase cleavage during apoptosis: When and where?. Exp. Cell Res..

[52] Bavetsias V., Linardopoulos S. (2015). Aurora kinase inhibitors: Current status and outlook. Front. Oncol..

[53] Du R., Huang C., Liu K., Li X., Dong Z. (2021). Targeting aurka in cancer: Molecular mechanisms and opportunities for cancer therapy. Mol. Cancer.

[54] Hecht M., Erber S., Harrer T., Klinker H., Roth T., Parsch H., Fiebig N., Fietkau R., Distel L.V. (2015). Efavirenz has the highest anti-proliferative effect of non-nucleoside reverse transcriptase inhibitors against pancreatic cancer cells. PLoS ONE.

[55] Fattore L., Malpicci D., Milite C., Castellano S., Sbardella G., Botti G., Ascierto P.A., Mancini R., Ciliberto G. (2020). Reverse transcriptase inhibition potentiates target therapy in braf-mutant melanomas: Effects on cell proliferation, apoptosis, DNA-damage, ros induction and mitochondrial membrane depolarization. Cell Commun. Signal..

[56] Perna A., Lucariello A., Sellitto C., Agliata I., Carleo M.A., Sangiovanni V., Esposito V., Guerra G., Cobellis L., De Luca A. (2017). Different cell cycle modulation in skov-3 ovarian cancer cell line by anti-hiv drugs. Oncol. Res..

[57] Chow W.A., Jiang C., Guan M. (2009). Anti-hiv drugs for cancer therapeutics: Back to the future?. Lancet Oncol..

[58] Mitsuyasu R.T. (2000). Aids-related kaposi’s sarcoma: Current treatment options, future trends. Oncology.

[59] Xiao B., Si H., Cervini A., Gao J., Lu J., Upadhyay S., Verma S., Robertson E. (2012). Kaposi’s sarcoma herpesvirus upregulates aurora a expression to promote p53 phosphorylation and ubiquitylation. PLoS Pathog..

[60] Zhu Q., Ding L., Zi Z., Gao S., Wang C., Wang Y., Zhu C., Yuan Z., Wei F., Cai Q. (2019). Viral-mediated aurkb cleavage promotes cell segregation and tumorigenesis. Cell Rep..

[61] Johnson J.R., Crosby D.C., Hultquist J.F., Kurland A.P., Adhikary P., Li D., Marlett J., Swann J., Hüttenhain R., Verschueren E. (2022). Global post-translational modification profiling of hiv-1-infected cells reveals mechanisms of host cellular pathway remodeling. Cell Rep..

[62] Bertolini F., Sukhatme V.P., Bouche G. (2015). Drug repurposing in oncology—Patient and health systems opportunities. Nat. Rev. Clin. Oncol..

[63] Carpinelli P., Moll J. (2008). Aurora kinase inhibitors: Identification and preclinical validation of their biomarkers. Expert Opin. Ther. Targets.

