# Supplementary material for: Anti-Leukaemic Activity of Rilpivirine Is Mediated by Aurora A Kinase Inhibition
Source: Cancers (Basel). 2023 Feb 7;15(4):1044. doi: 10.3390/cancers15041044 (PMC9954146; doi:10.3390/cancers15041044)
Supplement: Supplementary file 1 [file cancers-15-01044-s001.zip › cancers-2149387-supplementary.pdf]

Supplementary Information

Anti-Leukaemic Activity of Rilpivirine Is Mediated by Aurora A Kinase Inhibition

Saiful Islam [1], Muhammed H. Rahaman [1], Mingfeng Yu [1], Benjamin Noll [1], Jennifer H. Martin [2], Shudong Wang [1] and Richard Head [1,*]

1 Drug Discovery and Development, Clinical and Health Sciences, University of South Australia, Adelaide, SA 5000, Australia

2 Centre for Human Drug Repurposing and Medicines Research, University of Newcastle, Newcastle, NSW 2305, Australia

* Correspondence: richard.head@unisa.edu.au

<u>Table of contents</u> **<u>Page</u>**

Table S1. kinome-wide selectivity of rilpivirine at 1 µM……………………………………….**S3**

Table S2. Genetic backgrounds and French-American-British (FAB) subtypes of tested cell lines……………………………………………………………………………………..**S10**

Table S3. Pharmacokinetic properties of rilpivirine in mice…………………………………..**S13**

Figure S1. Effect of rilpivirine on the formation of colonies of HL-60 and NB4 cells………..**S10**

Figure S2. Cell cycle analysis of HL-60 cells following rilpivirine treatment (10 µM) at indicated time points……………………………………………………………………....**S11**

Figure S3. Plasma concentration-time profile of rilpivirine in mice after a single oral dose of 50 mg/kg (A) or a single IV dose of 2 mg/kg (B)…………………………………………………**S12**

Figure S4. Effect of rilpivirine on body weight of mice……………………………………….**S13**

Figure S5. *In vivo* toxicity assessment of rilpivirine in mice…………………………………..**S14**

Figure S6. Western blot analysis of HL-60 cells treated with rilpivirine, cytarabine or the combination at indicated concentrations for 72 hours………………………………………… **S15**

Figure S7. PIM1 expression across a panel of myeloid leukaemia cell lines shown by western blot analysis of cell lysate…………………………………………………………………....**S15**

Figure S8. (A) Dose-response curve for AZD1208 against K-562 cells at 72 hours. (B) Cell cycle analysis of K-562 cells after incubation with AZD1208 (60 µM) for 48 hours………….**S16**

Figure S9. Cell cycle analysis of KG1 cells following treatment with 10 µM or 20 µM rilpivirine for 24 hours …………………………………………………………………………………**S17**

Figure S10. Cell cycle analysis of NB4, U-937 and K-562 cells following treatment with 10 µM etravirine for 24 hours………………………………………………………………………… **S18**

Figure S11. Effect of rilpivirine on apoptosis of MV4-11 cells…………………………… **S19**

Figure S12. Original images of western blots for Figure 5A, left panel (HL-60)……………..**S20**

Figure S13. Original images of western blots for Figure 5A, left panel (HL-60) (continued)...**S21**

Figure S14. Original images of western blots for Figure 5A, right panel (NB4)……………...**S22**

Figure S15. Original images of western blots for Figure 5A, right panel (NB4) (continued)....**S23**

Figure S16. Original images of western blots for Figure 5B (HL-60 time course)…………....**S24**

Figure S17. Original images of western blots for Supplementary Figure S6………………….**S25**

Figure S18. Original images of western blots for Supplementary Figure S7………………….**S25**

Table S1. Kinome-wide selectivity of rilpivirine at 1 μM

Kinase	% Residual Activity	Kinase	% Residual Activity
AAK1(h)	87	BrSK2(h)	80
Abl(h)	86	BTK(h)	90
Abl(m)	63	BTK(R28H)(h)	97
Abl (H396P) (h)	71	B-Raf(h)	80
Abl (M351T)(h)	74	B-Raf(V599E)(h)	95
Abl (Q252H) (h)	94	CaMKI(h)	92
Abl(T315I)(h)	93	CaMKIß(h)	92
Abl(Y253F)(h)	58	CaMKIγ(h)	95
ACK1(h)	97	CaMKIIα(h)	61
ACTR2(h)	91	CaMKIIβ(h)	69
ALK(h)	80	CaMKIIγ(h)	58
ALK1(h)	84	CaMKIδ(h)	100
ALK2(h)	91	CaMKIIδ(h)	47
ALK4(h)	79	CaMKIV(h)	81
ALK6(h)	83	CaMKK1(h)	90
Arg(h)	70	CaMKK2(h)	109
AMPKα1(h)	44	Cdc7/cyclinB1(h)	93
AMPKα2(h)	48	CDK1/cyclinB(h)	105
A-Raf(h)	101	CDK2/cyclinA(h)	87
Arg(m)	48	CDK2/cyclinE(h)	92
ARK5(h)	82	CDK3/cyclinE(h)	90
ASK1(h)	92	CDK4/cyclinD3(h)	101
Aurora-A(h)	14	CDK5/p25(h)	97
Aurora-B(h)	85	CDK5/p35(h)	97
Aurora-C(h)	93	CDK6/cyclinD3(h)	96
Axl(h)	98	CDK7/cyclinH/MAT1(h)	78
BIKe(h)	56	CDK9/cyclin T1(h)	81
Blk(h)	73	CDK12/cyclinK(h)	85
Blk(m)	75	CDK13/cyclinK(h)	82
BMPR2(h)	96	CDK14/cyclinY(h)	91
Bmx(h)	98	CDK16/cyclinY(h)	96
BRK(h)	37	CDK17/cyclinY(h)	78
BrSK1(h)	84	CDK18/cyclinY(h)	93

Kinase	% Residual Activity	Kinase	% Residual Activity
CDKL1(h)	75	DCAMKL2(h)	122
CDKL2(h)	100	DCAMKL3(h)	95
CDKL3(h)	58	DDR1(h)	46
CDKL4(h)	74	DDR2(h)	82
ChaK1(h)	94	DMPK(h)	100
CHK1(h)	100	DRAK1(h)	76
CHK2(h)	93	DRAK2(h)	107
CHK2(I157T)(h)	97	DYRK1A(h)	56
CHK2(R145W)(h)	76	DYRK1B(h)	72
CK1α(h)	103	DYRK2(h)	89
CK1ε(h)	116	DYRK3(h)	84
CK1γ1(h)	80	eEF-2K(h)	92
CK1γ2(h)	90	EGFR(h)	111
CK1γ3(h)	107	EGFR(L858R)(h)	90
CK1δ(h)	99	EGFR(L861Q)(h)	35
CK1(y)	92	EGFR(T790M)(h)	79
CK2(h)	90	EGFR(T790M,L858R)(h)	88
CK2α1(h)	101	EphA1(h)	56
CK2α2(h)	104	EphA2(h)	72
CLIK1(h)	85	EphA3(h)	86
CLK1(h)	48	EphA4(h)	93
CLK2(h)	60	EphA5(h)	79
CLK3(h)	93	EphA7(h)	96
CLK4(h)	37	EphA8(h)	60
cKit(h)	99	EphB2(h)	78
cKit(D816V)(h)	88	EphB1(h)	94
cKit(D816H)(h)	89	EphB3(h)	88
cKit(V560G)(h)	74	EphB4(h)	86
CRIK(h)	107	ErbB2(h)	116
CSK(h)	51	ErbB4(h)	88
c-RAF(h)	91	FAK(h)	86
cSRC(h)	53	Fer(h)	91
DAPK1(h)	83	Fes(h)	85
DAPK2(h)	80	FGFR1(h)	94
DCAMKL1(h)	91	FGFR1(V561M)(h)	97

Kinase	% Residual Activity	Kinase	% Residual Activity
FGFR2(h)	95	IKKβ(h)	95
FGFR2(N549H)(h)	91	IKKε(h)	82
FGFR3(h)	98	IR(h)	111
FGFR4(h)	121	IR(h), activated	85
Fgr(h)	37	IRE1(h)	100
Flt1(h)	100	IRR(h)	95
Flt3(D835Y)(h)	94	IRAK1(h)	84
Flt3(h)	123	IRAK4(h)	80
Flt4(h)	101	Itk(h)	91
Fms(h)	91	JAK1(h)	79
Fms(Y969C)(h)	83	JAK2(h)	72
Fyn(h)	49	JAK3(h)	68
GCK(h)	99	JNK1α1(h)	90
GCN2(h)	93	JNK2α2(h)	85
GRK1(h)	103	JNK3(h)	100
GRK2(h)	81	KDR(h)	85
GRK3(h)	92	LATS1(h)	86
GRK5(h)	96	LATS2(h)	80
GRK6(h)	97	Lck(h)	33
GRK7(h)	70	Lck(h) activated	54
GSK3α(h)	80	LIMK1(h)	47
GSK3β(h)	84	LIMK2(h)	71
Haspin(h)	61	LKB1(h)	87
Hck(h)	50	LOK(h)	101
Hck(h) activated	63	Lyn(h)	23
HIPK1(h)	97	Lyn(m)	25
HIPK2(h)	85	LRRK2(h)	93
HIPK3(h)	92	LTK(h)	78
HIPK4(h)	87	MAK(h)	84
HPK1(h)	62	MAPK1(h)	90
HRI(h)	91	MAPK2(h)	101
ICK(h)	99	MAPK2(m)	92
IGF-1R(h)	89	MAP4K3(h)	91
IGF-1R(h), activated	99	MAP4K4(h)	79
IKKα(h)	85	MAP4K5(h)	34

Kinase	% Residual Activity	Kinase	% Residual Activity
MAPKAP-K2(h)	100	MST1(h)	84
MAPKAP-K3(h)	90	MST2(h)	80
MEK1(h)	101	MST3(h)	93
MEK2(h)	92	MST4(h)	91
MARK1(h)	82	mTOR(h)	97
MARK3(h)	87	mTOR/FKBP12(h)	100
MARK4(h)	88	MuSK(h)	96
MEKK2(h)	104	MYLK2(h)	79
MEKK3(h)	72	MYO3B(h)	96
MELK(h)	56	NDR1(h)	89
Mer(h)	74	NDR2(h)	100
Met(h)	80	NEK1(h)	86
Met(D1246H)(h)	95	NEK2(h)	97
Met(D1246N)(h)	102	NEK4(h)	97
Met(M1268T)(h)	109	NEK3(h)	91
Met(Y1248C)(h)	100	NEK6(h)	94
Met(Y1248D)(h)	100	NEK7(h)	110
Met(Y1248H)(h)	98	NEK9(h)	90
MINK(h)	80	NIM1(h)	88
MKK3(h)	111	NEK11(h)	92
MKK4(m)	84	NLK(h)	64
MKK6(h)	94	NUAK2(h)	101
MLCK(h)	95	OSR1(h)	102
MLK1(h)	81	p70S6K(h)	108
MLK2(h)	85	PAK1(h)	92
MLK3(h)	81	PAK2(h)	89
MLK4(h)	95	PAK4(h)	87
Mnk2(h)	85	PAK3(h)	86
MOK(h)	92	PAK5(h)	91
MRCKα(h)	86	PAK6(h)	117
MRCKβ(h)	87	PAR-1Bα(h)	104
MRCKγ(h)	90	PASK(h)	85
MSK1(h)	97	PEK(h)	82
MSK2(h)	105	PDGFRα(h)	96
MSSK1(h)	84	PDGFRα(D842V)(h)	79

Kinase	% Residual Activity	Kinase	% Residual Activity
PDGFRα(V561D)(h)	86	PRAK(h)	100
PDGFRβ(h)	93	PRKG2(h)	92
PDHK2(h)	90	PRK1(h)	64
PDHK4(h)	94	PRK2(h)	74
PDK1(h)	85	PrKX(h)	97
PhKγ1(h)	80	PRP4(h)	113
PhKγ2(h)	83	PTK5(h)	32
Pim-1(h)	22	Pyk2(h)	81
Pim-2(h)	56	Ret(h)	108
Pim-3(h)	89	Ret (V804L)(h)	91
PKA(h)	93	Ret(V804M)(h)	92
PKAcβ(h)	93	RIPK1(h)	91
PKBα(h)	80	RIPK2(h)	45
PKBβ(h)	90	ROCK-I(h)	95
PKBγ(h)	86	ROCK-II(h)	98
PKCα(h)	83	ROCK-II(r)	94
PKCβI(h)	89	Ron(h)	85
PKCβII(h)	94	Ros(h)	77
PKCγ(h)	103	Rse(h)	66
PKCδ(h)	72	Rsk1(h)	91
PKCε(h)	89	Rsk1(r)	90
PKCη(h)	103	Rsk2(h)	82
PKCι(h)	99	Rsk3(h)	86
PKCμ(h)	98	Rsk4(h)	99
PKCθ(h)	66	SAPK2a(h)	84
PKCζ(h)	80	SAPK2a(T106M)(h)	90
PKD2(h)	115	SAPK2b(h)	98
PKD3(h)	76	SAPK3(h)	106
PKG1α(h)	88	SAPK4(h)	97
PKG1β(h)	79	SBK1(h)	100
PKR(h)	92	SGK(h)	97
Plk1(h)	107	SGK2(h)	97
Plk3(h)	99	SGK3(h)	99
Plk4(h)	85	SIK(h)	66

Kinase	% Residual Activity	Kinase	% Residual Activity
SIK2(h)	57	TrkB(h)	72
SIK3(h)	89	TrkC(h)	91
SLK(h)	106	TSSK1(h)	86
Snk(h)	98	TSSK2(h)	91
SNRK(h)	104	TSSK3(h)	95
Src(1-530)(h)	31	TSSK4(h)	127
Src(T341M)(h)	78	TTBK1(h)	90
SRMS(h)	55	TTBK2(h)	88
SRPK1(h)	84	TTK(h)	71
SRPK2(h)	91	Txk(h)	79
STK16(h)	58	TYK2(h)	52
STK25(h)	95	ULK1(h)	85
STK32A(h)	89	ULK2(h)	90
STK32B(h)	99	ULK3(h)	94
STK32C(h)	94	VRK1(h)	80
STK33(h)	102	VRK2(h)	88
STK39(h)	95	Wee1(h)	90
Syk(h)	79	Wee1B(h)	94
TAF1L(h)	55	WNK1(h)	95
TAK1(h)	105	WNK2(h)	80
TAO1(h)	41	WNK3(h)	83
TAO2(h)	59	WNK4(h)	113
TAO3(h)	65	Yes(h)	17
TBK1(h)	72	ZAK(h)	99
Tec(h) activated	105	ZAP-70(h)	97
TGFBR1(h)	103	ZIPK(h)	85
TGFBR2(h)	94	ATM(h)	111
Tie2 (h)	93	ATR/ATRIP(h)	117
Tie2(R849W)(h)	86	DNA-PK(h)	95
Tie2(Y897S)(h)	96	PI3 Kinase (p110b/p85a)(h)	104
TLK1(h)	95	PI3 Kinase (p120g)(h)	87
TLK2(h)	115	PI3 Kinase (p110d/p85a)(h)	89
TNIK(h)	50	PI3 Kinase (p110a/p85a)(m)	100
TRB2(h)	65	PI3 Kinase (p110a/p65a)(m)	104
TrkA(h)	101	PI3 Kinase (p110a(E545K)/p85a)(m)	97

Kinase	% Residual Activity		
PI3 Kinase (p110a(H1047R)/p85a)(m)	96		
PI3 Kinase (p110b/p85b)(m)	97		
PI3 Kinase (p110b/p85a)(m)	98		
PI3 Kinase (p110d/p85a)(m)	99		
PI3 Kinase (p110a(E542K)/p85a)(m)	101		
PI3 Kinase (p110a/p85a)(h)	98		
PI3 Kinase (p110a(E542K)/p85a)(h)	97		
PI3 Kinase (p110aH1047R)/p85a)(h)	93		
PI3 Kinase (p110a(E545K)/p85a)(h)	98		
PI3 Kinase (p110a/p65a)(h)	93		
PI3KC2a(h)	94		
PI3KC2g(h)	82		
PIP4K2a(h)	103		
PIP5K1a(h)	96		
PIP5K1g(h)	76		

Table S2. Genetic backgrounds and French-American-British (FAB) subtypes of tested cell lines

Cell lines	Leukaemia type	FAB subtype	Oncogenic drivers	Ref
HL-60	AML	M2	N-RAS	[24,25]
NB4	AML	M3	PML-RARA	
K-562	CML	M1	BCR-ABL	
U-937	AML	M5	MLLT10-PICALM	

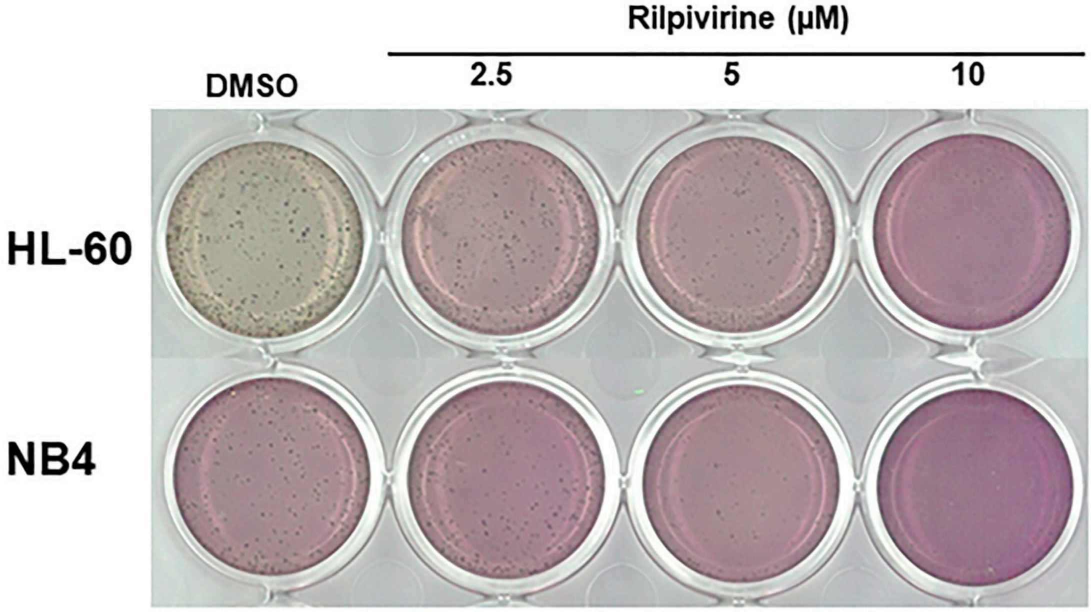

Figure S1. Effect of rilpivirine on the formation of colonies of HL-60 and NB4 cells. Cells were treated with rilpivirine at indicated concentrations. After 10 days, cells were stained with MTT and colonies were observed in an inverted microscope. Representative images are shown.

HL-60

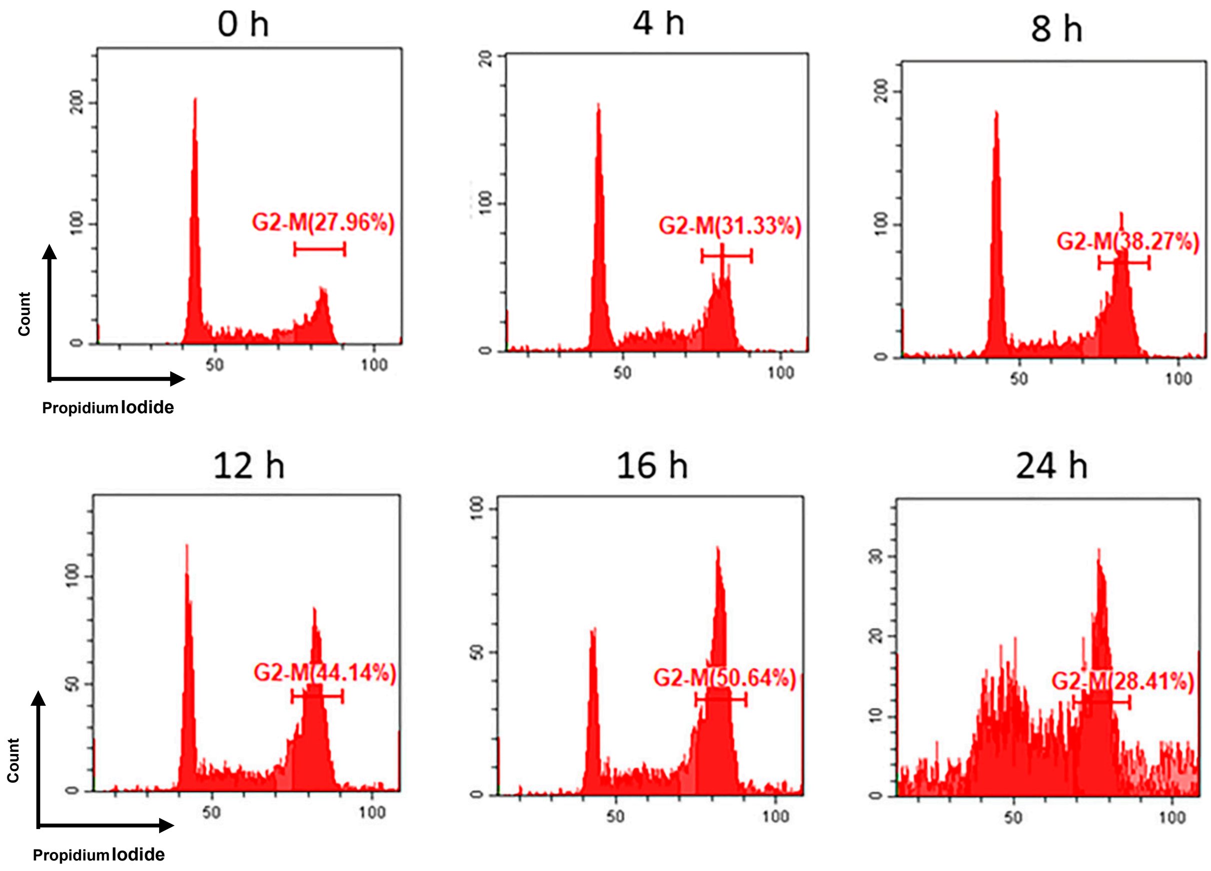

Figure S2. Cell cycle analysis of HL-60 cells following rilpivirine treatment (10 μM) at indicated time points. Representative plots from flow cytometry are shown.

A

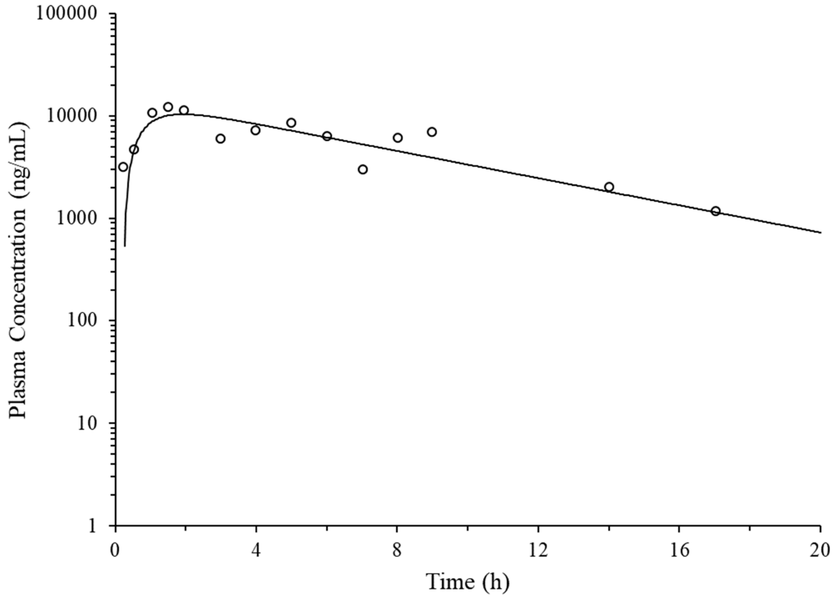

B

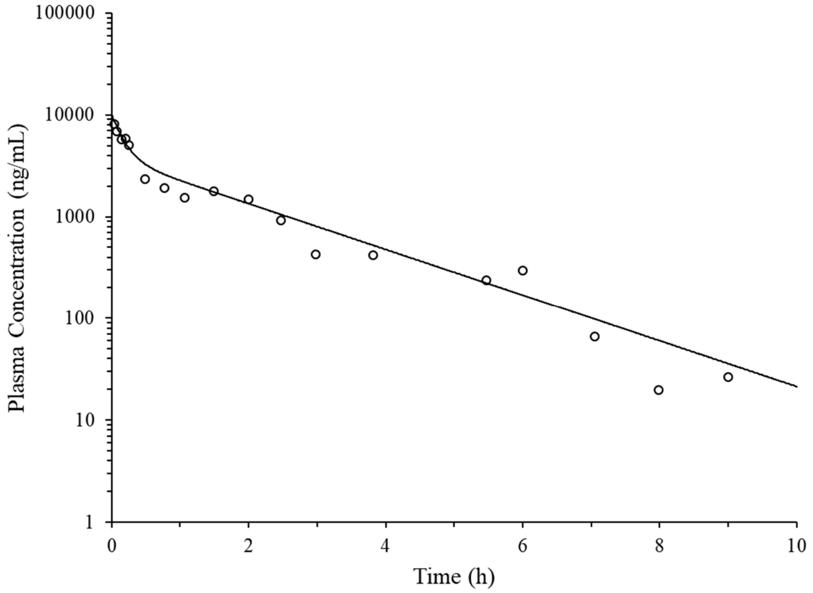

Figure S3. Plasma concentration-time profile of rilpivirine in mice after a single oral dose of 50 mg/kg (A) or a single IV dose of 2 mg/kg (B).

Table S3. Pharmacokinetic properties of rilpivirine in mice.

Pharmacokinetic properties of rilpivirine in mice						
	Parameter					
**Route**	**Dose (mg/kg)**	**C_{max} (μM)**	**AUC_{0-t} (μM*h)**	**$t_{1/2}$ (h)**	**T_{max} (h)**	***F* (%)**
**PO**	50.00	33.30	247.3	4.91	1.50	49.1
**IV**	2.00	22.40	20.0	1.30	0.033	-

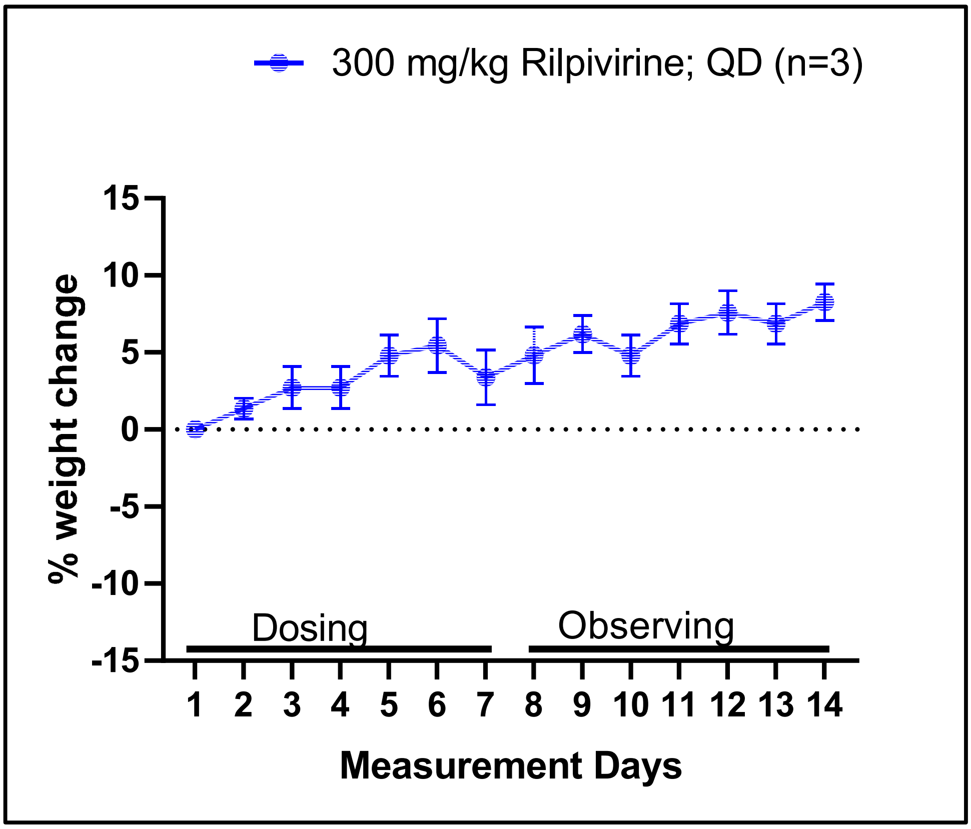

Figure S4. Effect of rilpivirine on body weight of mice. Female nude mice (n = 3 per group) were administered oral doses of rilpivirine at 300 mg/kg per day for seven days followed by seven days of observation. Body weight was assessed daily. Data were presented as mean ± SEM.

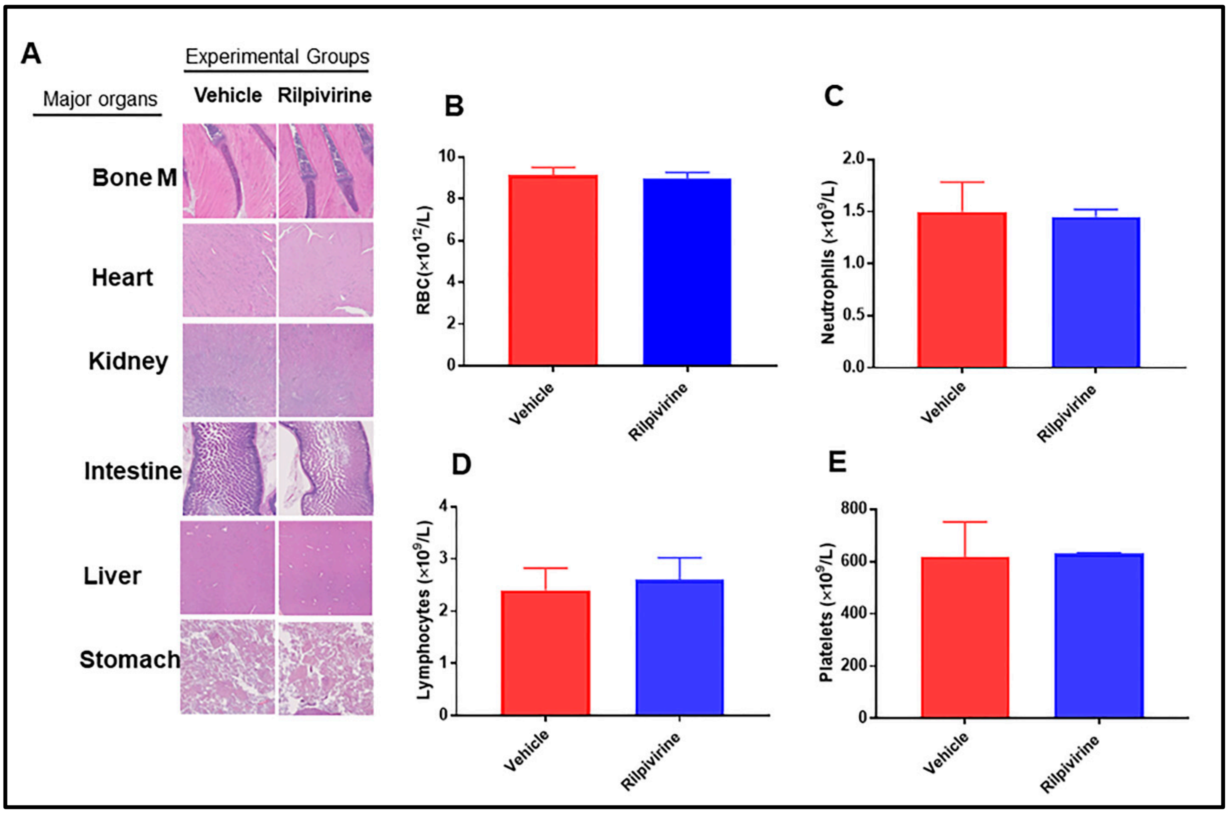

Figure S5. ***In vivo*** **toxicity assessment of rilpivirine in mice:** HL-60 tumour-bearing mice (n = 3) were treated with vehicle (0.5% HPMC, PO, QD) or rilpivirine (200 mg/kg, PO, QD) for 14 days. Blood, bone marrow, heart, kidney, intestine, liver and stomach were collected 24 hours post final dose. (A) Histopathological study of bone marrow, heart, kidney, intestine, liver and stomach. Complete blood count analysis of (B) red blood cells, (C) neutrophils, (D) lymphocytes, and (E) platelets 24 hours post final treatment.

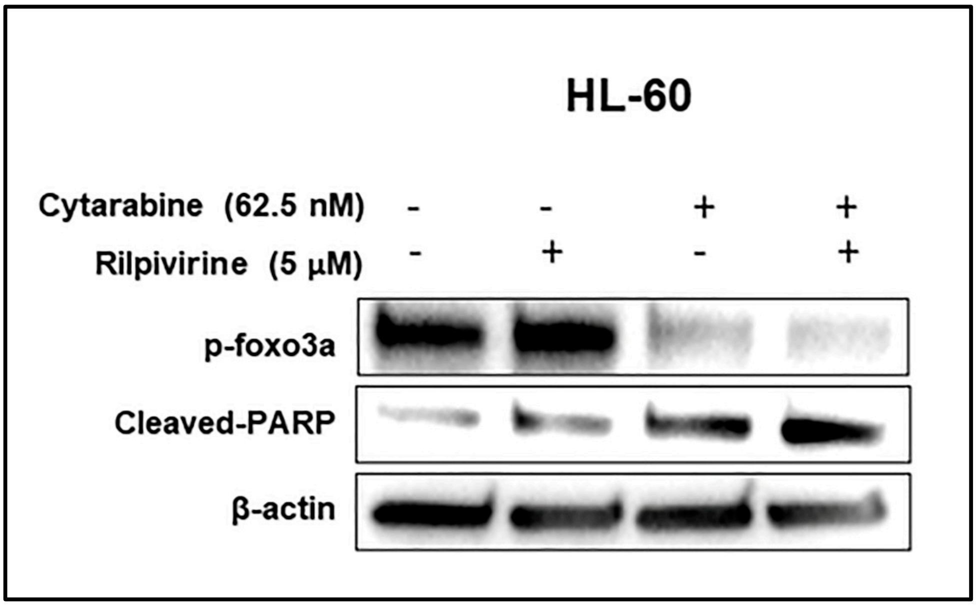

Figure S6. Western blot analysis of HL-60 cells treated with rilpivirine, cytarabine, or the combination at indicated concentrations for 72 hours. Antibodies used are indicated on the left side of each panel, respectively. DMSO diluent is used as a control and β-actin used as an internal loading control.

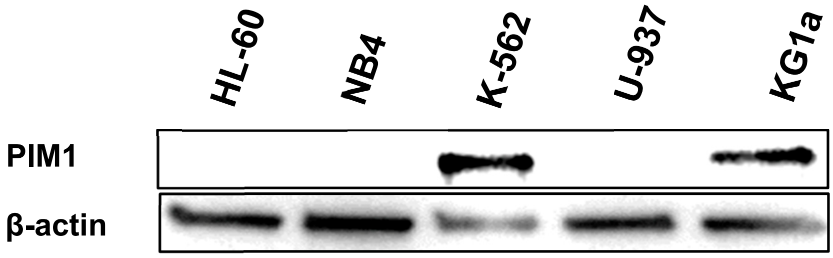

Figure S7. PIM1 expression across a panel of myeloid leukaemia cell lines shown by western blot analysis of cell lysates. β-Actin was used as an internal loading control. KG1a cell line was used as a positive control.

A

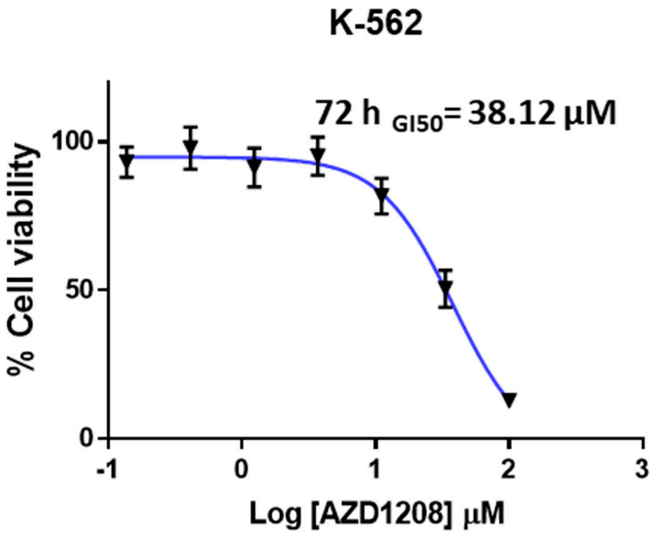

B

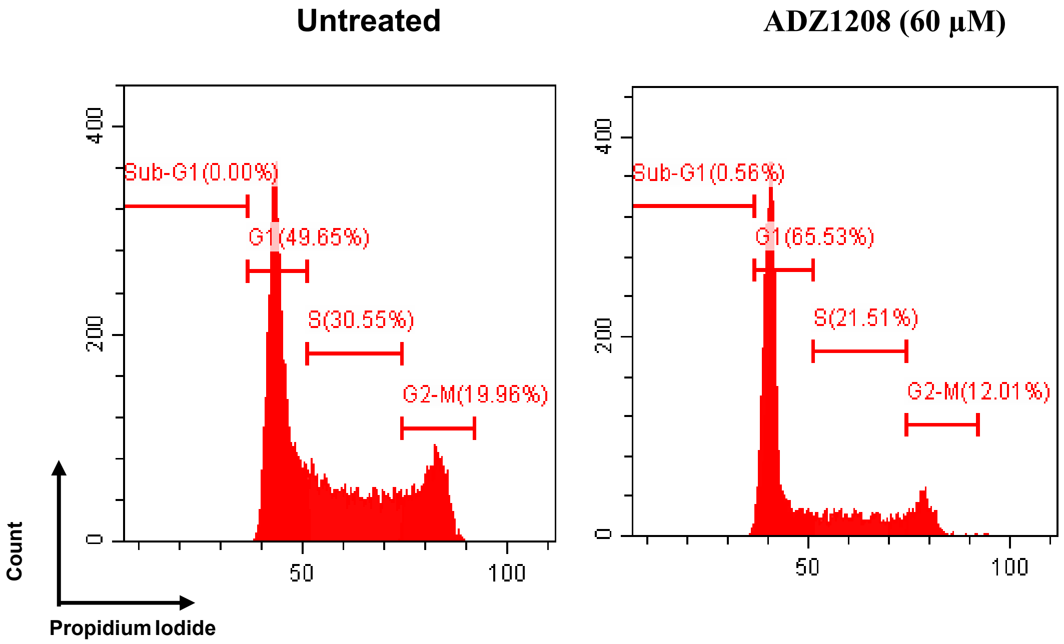

Figure S8. (A) Dose-response curve for AZD1208 against K-562 cells at 72 hours. GI_{50} value (µM) is shown. (B) Cell cycle analysis of K-562 cells after incubation with AZD1208 (60 µM) for 48 hours. Representative histograms with DNA content are shown.

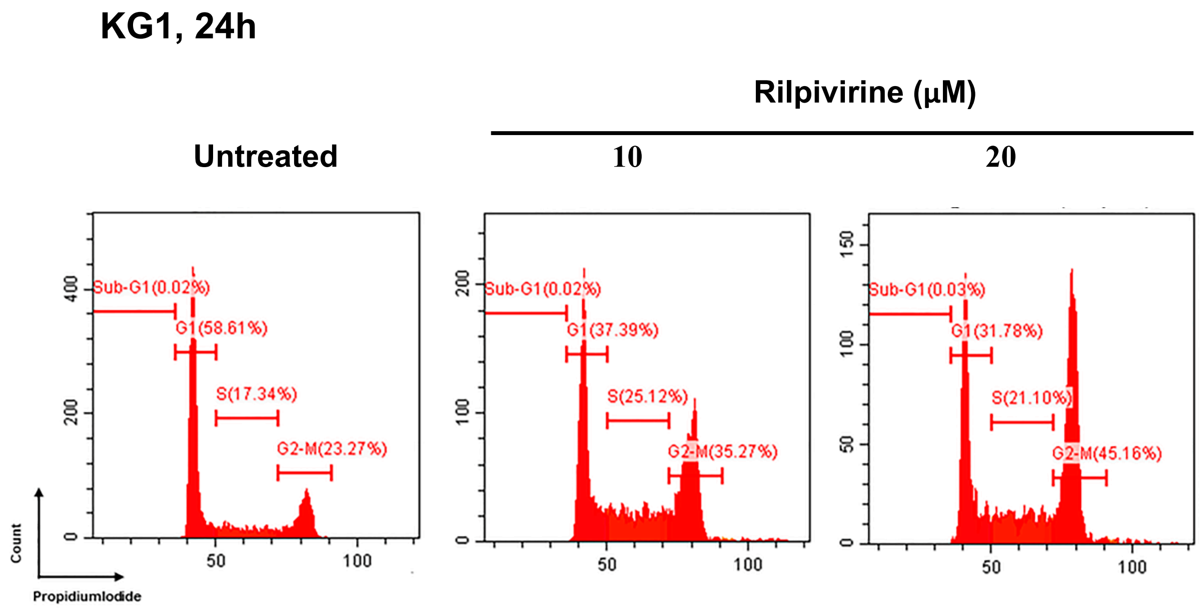

Figure S9. Cell cycle analysis of KG1 cells following treatment with 10 µM or 20 µM rilpivirine for 24 hours. Representative histograms with DNA content are shown.

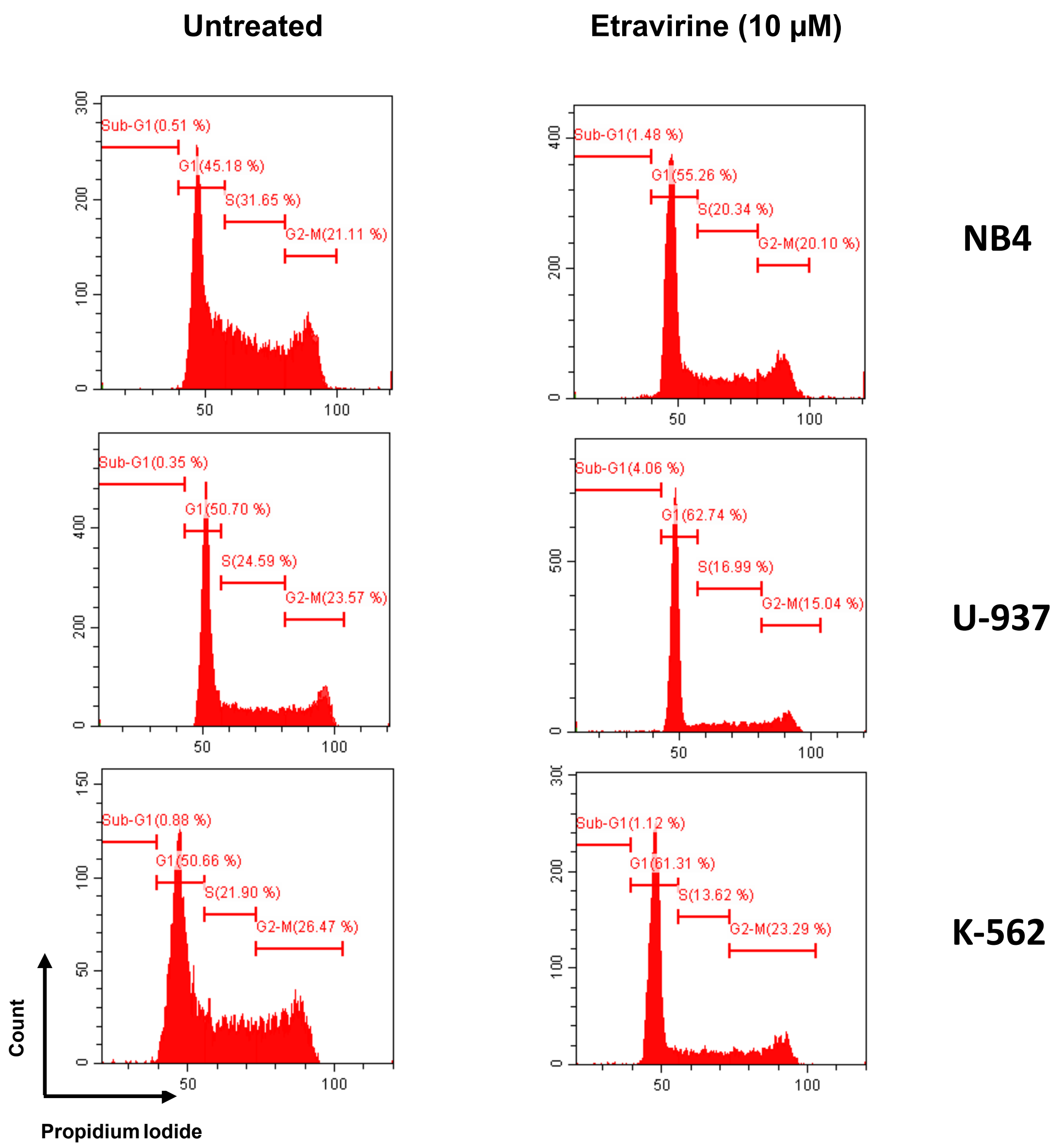

Figure S10. Cell cycle analysis of NB4, U-937 and K-562 cells following treatment with 10 µM etravirine for 24 hours. Representative histograms with DNA content are shown.

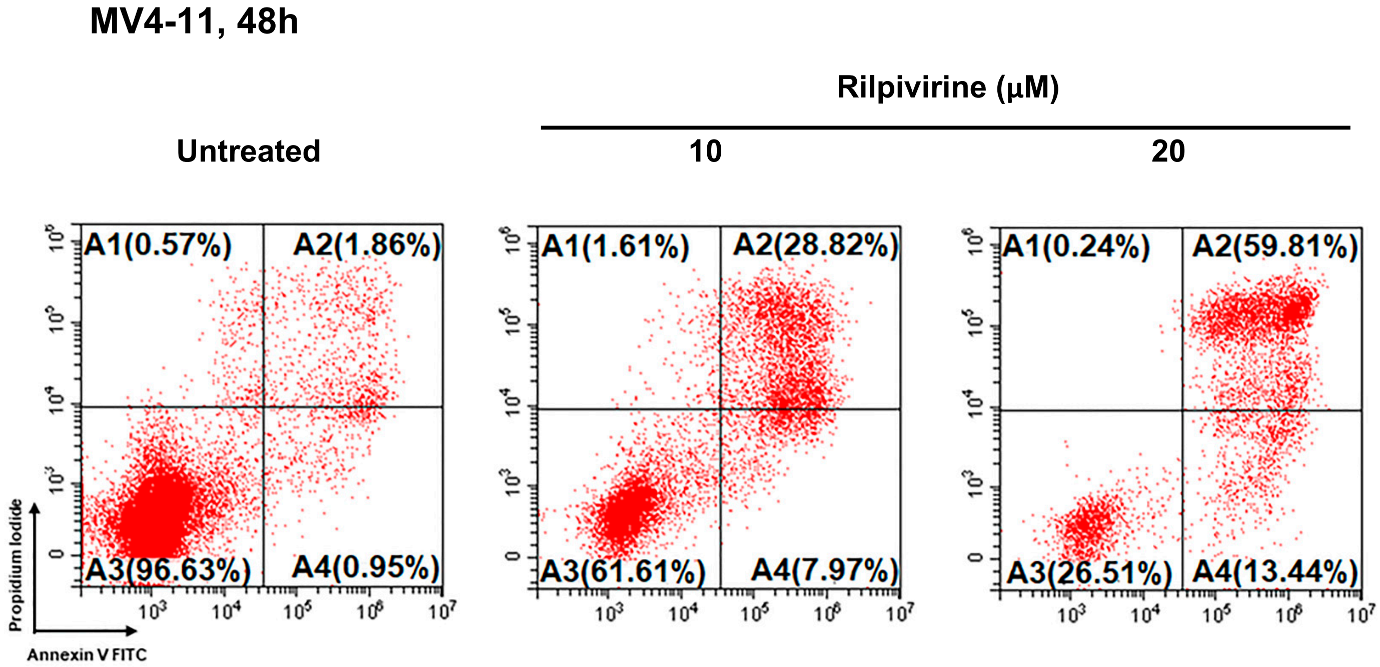

Figure S11. Effect of rilpivirine on apoptosis of MV4-11 cells. MV4-11 cells were treated with 10 or 20 µM of rilpivirine for 48 hours and apoptosis was detected using annexin V/PI double staining. Representative figures from flow cytometric analysis are shown.

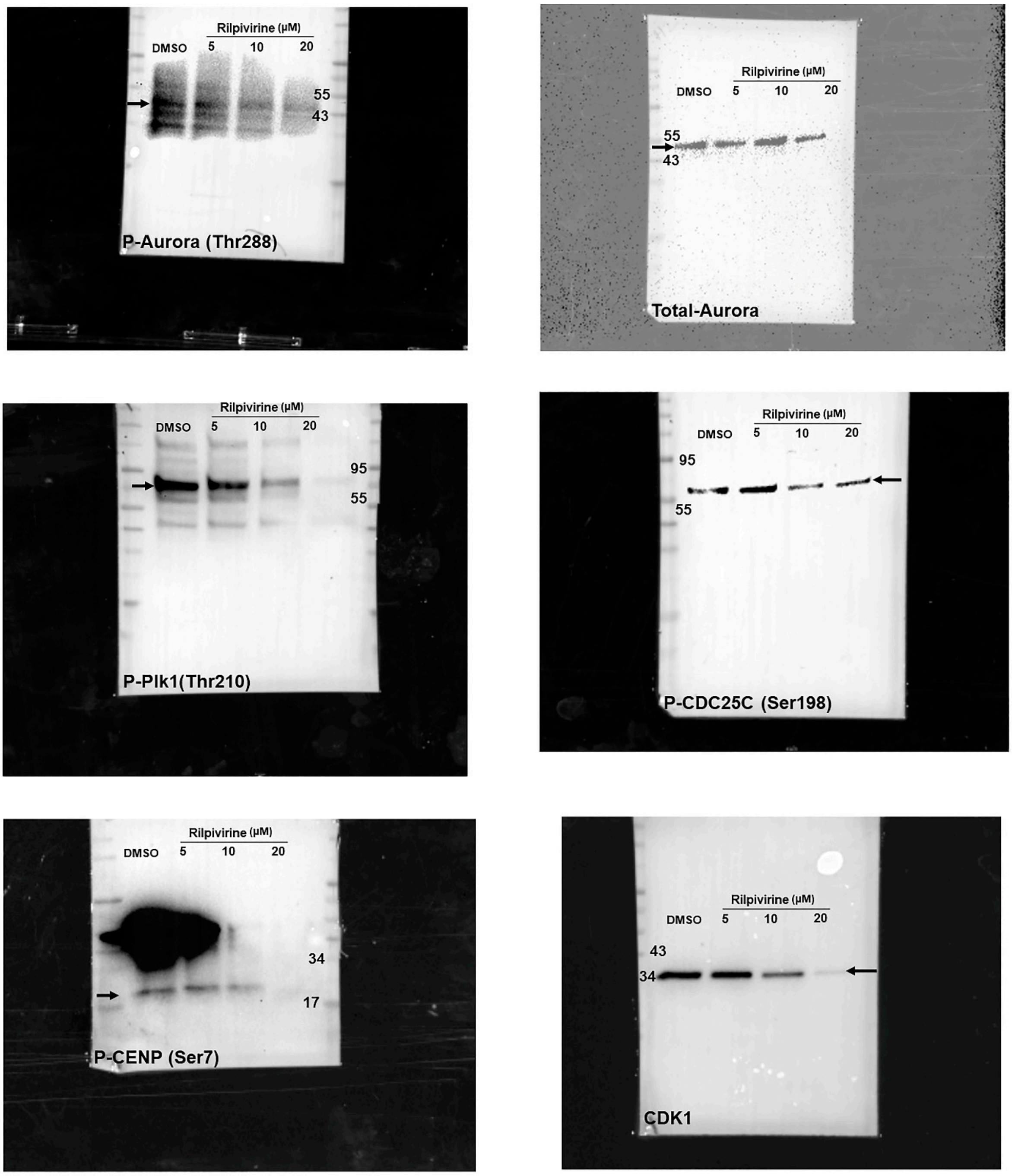

Figure S12. Original images of western blots for Figure 5A, left panel (HL-60).

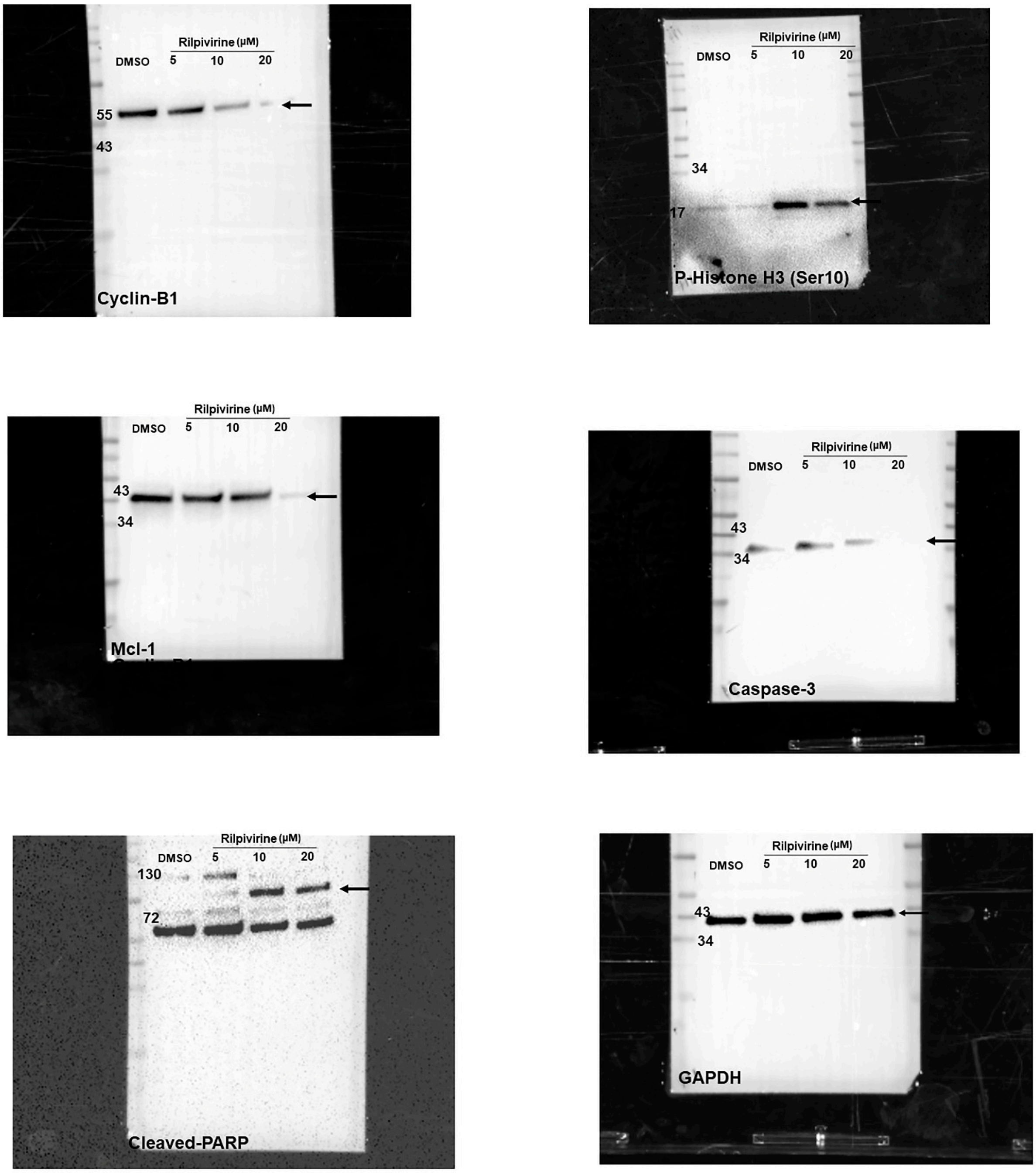

Figure S13. Original images of western blots for Figure 5A, left panel (HL-60) (continued).

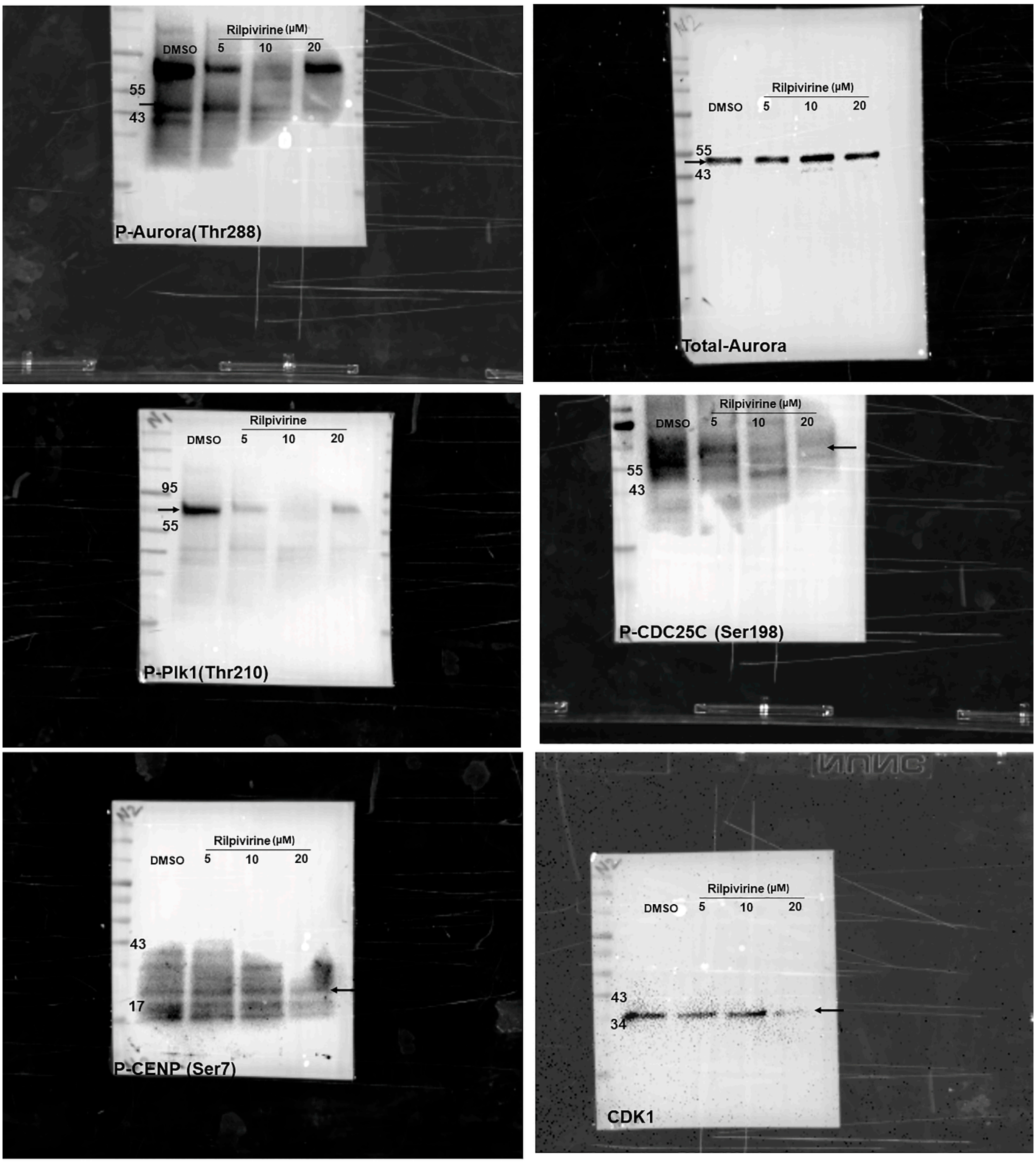

Figure S14. Original images of western blots for Figure 5A, right panel (NB4).

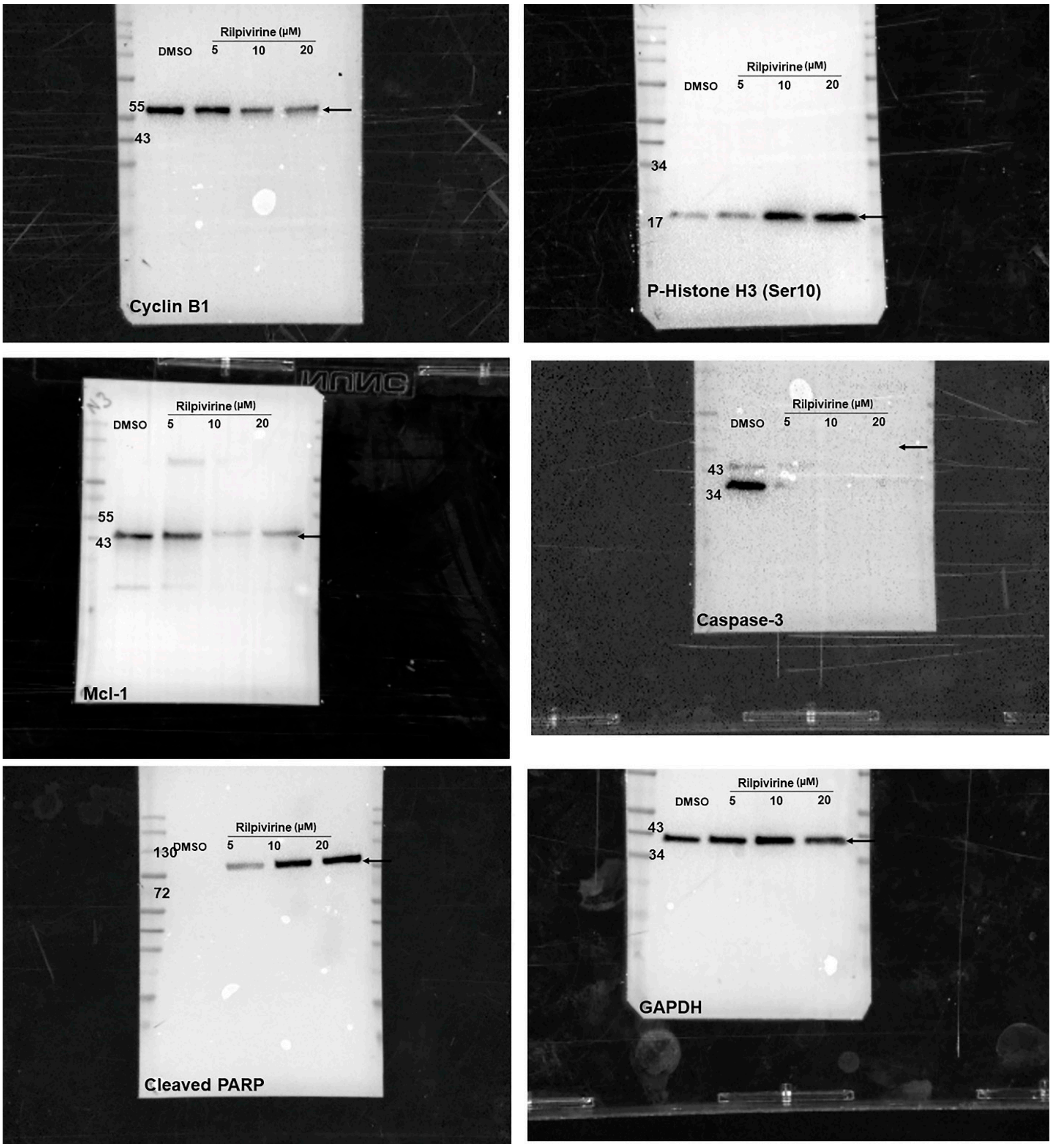

Figure S15. Original images of western blots for Figure 5A, right panel (NB4) (continued).

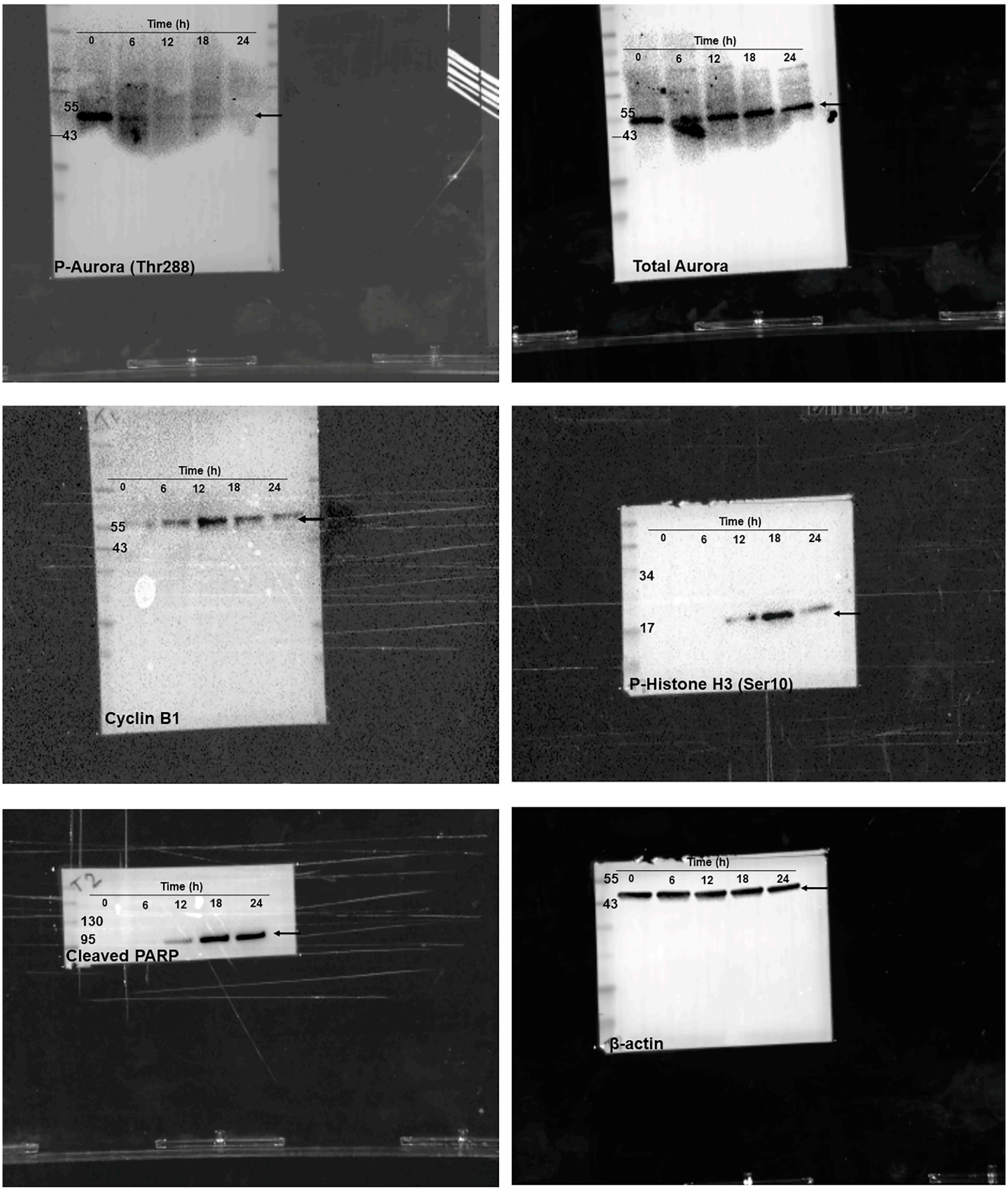

Figure S16. Original images of western blots for Figure 5B (HL-60 time course).

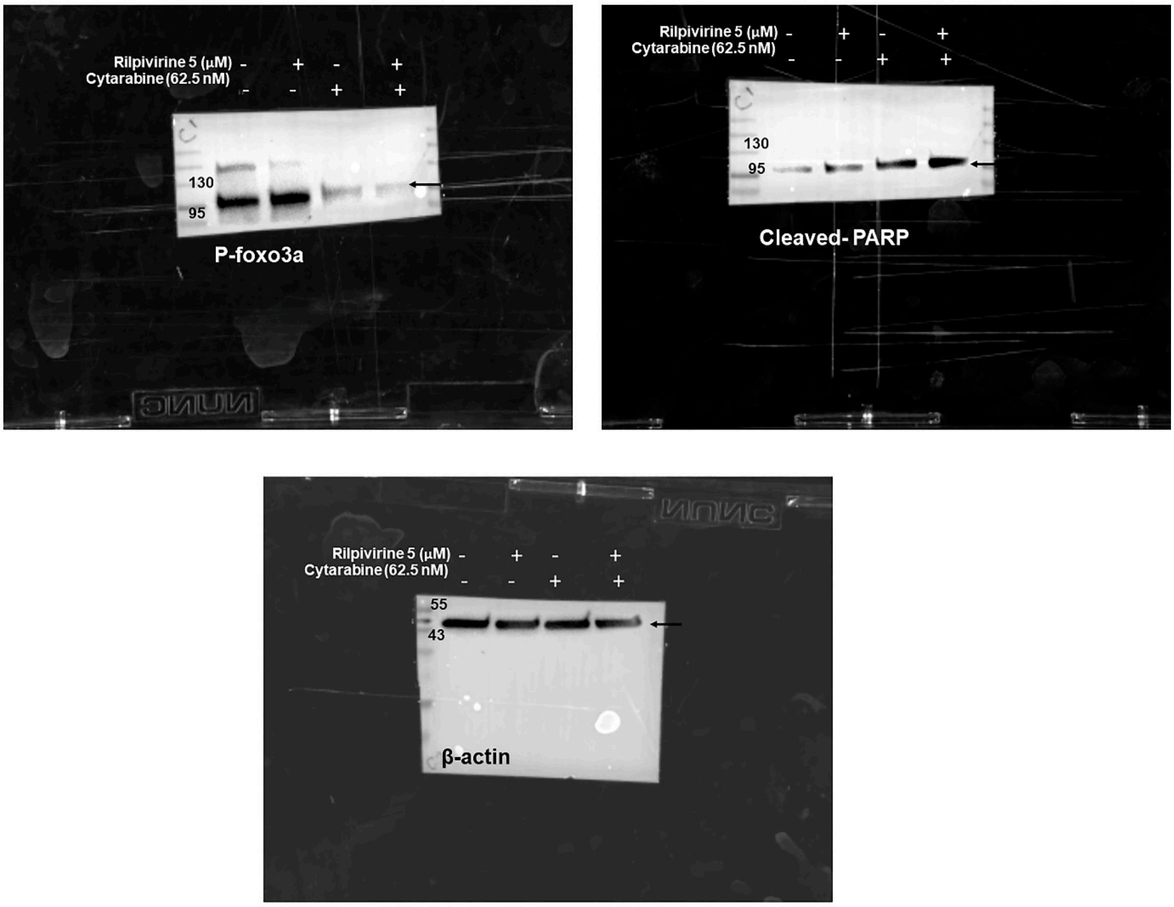

Figure S17. Original images of western blots for Supplementary Figure S6.

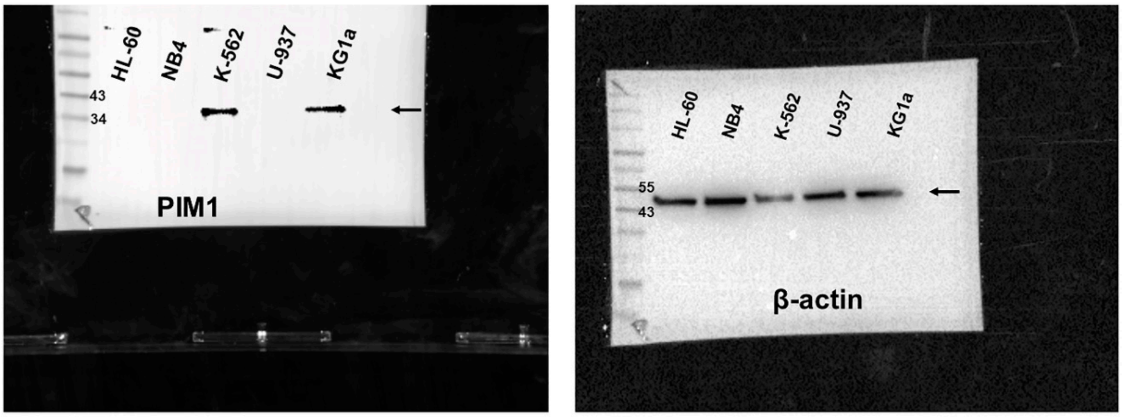

Figure S18. Original images of western blots for Supplementary Figure S7.
